# *Chlamydomonas reinhardtii*, *Volvox carteri* and related green algae accumulate ketocarotenoids not in vegetative cells but in zygospores

**DOI:** 10.1111/tpj.17261

**Published:** 2025-02-09

**Authors:** Sonja Schwarz, Matthias Bauch, Volker Schmitt, Armin Hallmann, Martin Lohr

**Affiliations:** ^1^ Institut für Molekulare Physiologie Johannes Gutenberg‐Universität 55099 Mainz Germany; ^2^ Zell‐ und Entwicklungsbiologie der Pflanzen Universität Bielefeld Universitätsstr. 25 33615 Bielefeld Germany

**Keywords:** *Chlamydomonas reinhardtii*, *Volvox carteri*, *Chlamydomonadales*, *zygospore*, *ketocarotenoids*, *astaxanthin*, *4‐ketolutein*, *BKT*, *thylakoid breakdown*, *storage lipids*

## Abstract

Zygospores of green alga such as *Chlamydomonas reinhardtii*, *Volvox carteri* or *Dunaliella salina* display a bright orange color indicative of carotenoids, yet there have been no reports on their pigment composition. The genomes of these algae contain genes for homologs of the β‐carotene ketolase (BKT) from the well‐known astaxanthin producer *Haematococcus pluvialis*, that were assumed to be pseudogenes, because none of these species has been reported to accumulate astaxanthin or other ketocarotenoids. Here, we show that *C. reinhardtii* and *V. carteri* synthesize ketocarotenoids specifically in zygospores. Contrary to the vegetative aplanospores of *H. pluvialis*, the major ketocarotenoid in zygospores of *C. reinhardtii* is not astaxanthin but 4‐ketolutein. Moreover, the ketocarotenoids in maturing zygospores are not synthesized de novo but from carotenoids of the photosynthetic apparatus liberated by a massive breakdown of thylakoid membranes. In line with this conclusion, incubation of zygospores at 9°C instead of 22°C resulted in a reduced thylakoid breakdown and only low amounts of ketocarotenoids, while the accumulation of storage lipids was less affected. Furthermore, we show the full‐length BKT from *C. reinhardtii* to catalyze the ketolation of both α‐carotene and lutein in carotenogenic bacteria. We also detected putative BKT genes in the genomes of various other green algae not yet known to synthesize ketocarotenoids, suggesting a zygospore‐specific accumulation of ketocarotenoids to be common among Chlamydomonadales. Our observation that zygospores of *C. reinhardtii* accumulate ketocarotenoids together with storage lipids sheds light on the physiology of a largely unexplored algal life stage crucial for survival and propagation.

## Significance Statement

While various green algae accumulate ketocarotenoids such as astaxanthin in the vegetative state, we found that the model *Chlamydomonas reinhardtii* synthesizes ketocarotenoids only in the diploid zygospores by recycling photosynthetic carotenoids released during breakdown of thylakoid membranes. Our observation explains the presence of carotene ketolase genes in genomes of algae such as *Chlamydomonas*, *Volvox*, and *Dunaliella* not known to accumulate ketocarotenoids, and enables exploiting the molecular toolbox for *Chlamydomonas* in research on algal ketocarotenoid metabolism.

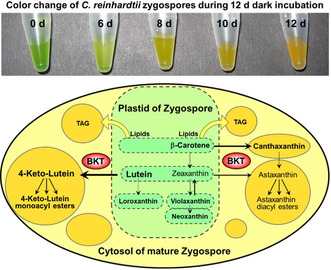

## INTRODUCTION

Ketocarotenoids are carotenoids containing at least one keto group, but the term is most commonly used for cyclic carotenoids with their β‐ionone ring(s) ketolated at position C4. The most prominent ketocarotenoids are canthaxanthin (4,4′‐diketo‐β‐carotene) and astaxanthin (3,3′‐dihydroxy‐4,4′‐diketo‐β‐carotene), which are synthesized by various bacteria, algae, fungi, and some non‐photosynthetic protists (Fang et al., 2019; Misawa, 2009) but absent from land plants, with the exception of flowers from some species of the genus *Adonis* (Cunningham & Gantt, 2005; Renstrom et al., 1981). Astaxanthin and canthaxanthin are also encountered in many aquatic animals, as for example crustaceans (lobster, shrimp) or fish (salmon, trout), and water birds like flamingo (Goodwin, 1984). These animals, however, are not capable of synthesizing carotenoids but instead rely on their uptake by food. Astaxanthin and canthaxanthin are also of ever‐growing commercial interest due to their coloration and antioxidant properties (Nair et al., 2023; Rebelo et al., 2020). The major market for astaxanthin is aquaculture where it is used as food additive for fish farming, but there is also a growing nutraceutical and pharmaceutical market (Ambati et al., 2014; Camacho et al., 2019; Nair et al., 2023). Currently, astaxanthin is supplied mainly by chemical synthesis (Zhang et al., 2020); however, biological production by microorganisms, like the yeast *Xanthophyllomyces dendrorhous* (formerly *Phaffia rhodozyma*) and the green alga *Haematococcus pluvialis*, has been the focus of intense studies, and *H. pluvialis* is a major source of biogenic (natural) astaxanthin (Oslan et al., 2021).

In addition to *H. pluvialis*, ketocarotenoid accumulation has also been observed in the vegetative stages of other green algae, for example, in *Chromochloris* (*Chlorella*) *zofingiensis*, the ice alga *Chlamydomonas nivalis* and various species of the genera *Ankistrodesmus*, *Chlorococcum* and *Scenedesmus* (Czygan, 1968; Goodwin, 1980; Zhang et al., 2021). It usually is induced by unfavorable nutrient conditions and is often coupled to the development of vegetative resting stages with reinforced cell walls that accumulate significant amounts of storage lipids (Boussiba, 2000). The lipid droplets contain the lipophilic ketocarotenoids, which are mostly esterified with fatty acids that make them even more hydrophobic (Holtin et al., 2009). The enzyme β‐carotene ketolase (BKT) is a key enzyme in the formation of ketocarotenoids in green algae and was first identified in *H. pluvialis*. By heterologous expression in carotenogenic bacteria (Kajiwara et al., 1995; Lotan & Hirschberg, 1995) and by *in vitro* assays (Fraser et al., 1997), the BKT from *H. pluvialis* was shown to add keto groups at C4 of the β‐ionone rings in β‐carotene or zeaxanthin yielding canthaxanthin or astaxanthin, respectively.

In the green alga *Chlamydomonas reinhardtii*, no ketocarotenoids have been detected despite more than 70 years of work on photosynthesis and pigments in this well‐established model organism (reviewed in Lohr, 2023). Analyses of the genome of *C. reinhardtii*, however, revealed the presence of a gene encoding a protein with 70% sequence identity to the BKT from *H. pluvialis* (Grossman et al., 2004; Lohr et al., 2005). The predicted gene product showed a significantly longer C‐terminus than the BKT enzymes from *H. pluvialis* and other green algae, which led to speculations that this extension may render the enzyme inactive (Lohr et al., 2005). Heterologous expression of a truncated version of the BKT from *C. reinhardtii* without the C‐terminal 116 amino acids in non‐photosynthetic carotenogenic bacteria, *Arabidopsis thaliana*, tobacco, or tomato resulted in a functional enzyme that was competent of ketolating both β‐carotene and zeaxanthin (Huang et al., 2012, 2013; Zhong et al., 2011). Moreover, expression in *C. reinhardtii* of a codon‐optimized version of the truncated BKT gene resulted in a substantial accumulation of astaxanthin and canthaxanthin, prompting the suggestion that the endogenous BKT gene may be a pseudogene (Chen et al., 2023; Perozeni et al., 2020; Sharma et al., 2024). More recently, a BKT gene was also found in the genome of a strain of the green alga *Dunaliella salina* known for its ability to accumulate massive amounts of β‐carotene under stress conditions such as high salinity or high light (Chen et al., 2022). As with *C. reinhardtii*, the product of the BKT gene from *D. salina* was shown to be functional when expressed in carotenogenic bacteria (Chen et al., 2022), although no ketocarotenoids have been detected in *D. salina* so far.

Mature zygospores of *C. reinhardtii* have been reported to be orange‐colored with the color residing mainly in cytosolic lipid droplets (Cavalier‐Smith, 1976). This resembles observations on the resting spores of *H. pluvialis* (Grünewald et al., 2001; Santos & Mesquita, 1984) suggesting that in *C. reinhardtii* and other green algae, the ketolase activity and formation of ketocarotenoids might be confined to the diploid resting stage of the mature zygospores (hypnozygotes). This speculation led us to investigate the changes in the pigment composition and ultrastructure of maturing zygospores from *C. reinhardtii*. Furthermore, we searched for BKT genes in other green algal genomes and analyzed the pigment composition of zygospores of the multicellular green alga *Volvox carteri*, which is a close relative of *C. reinhardtii*. Our results indicate that the ability to accumulate ketocarotenoids is more widespread among green algae than assumed but has been overlooked in cases when it is confined to the zygospore stage, which is less amenable to experimental studies than the vegetative stage.

## RESULTS

### Mature zygospores of *C. reinhardtii* accumulate ketocarotenoids as the main pigments

**Figure 1:**
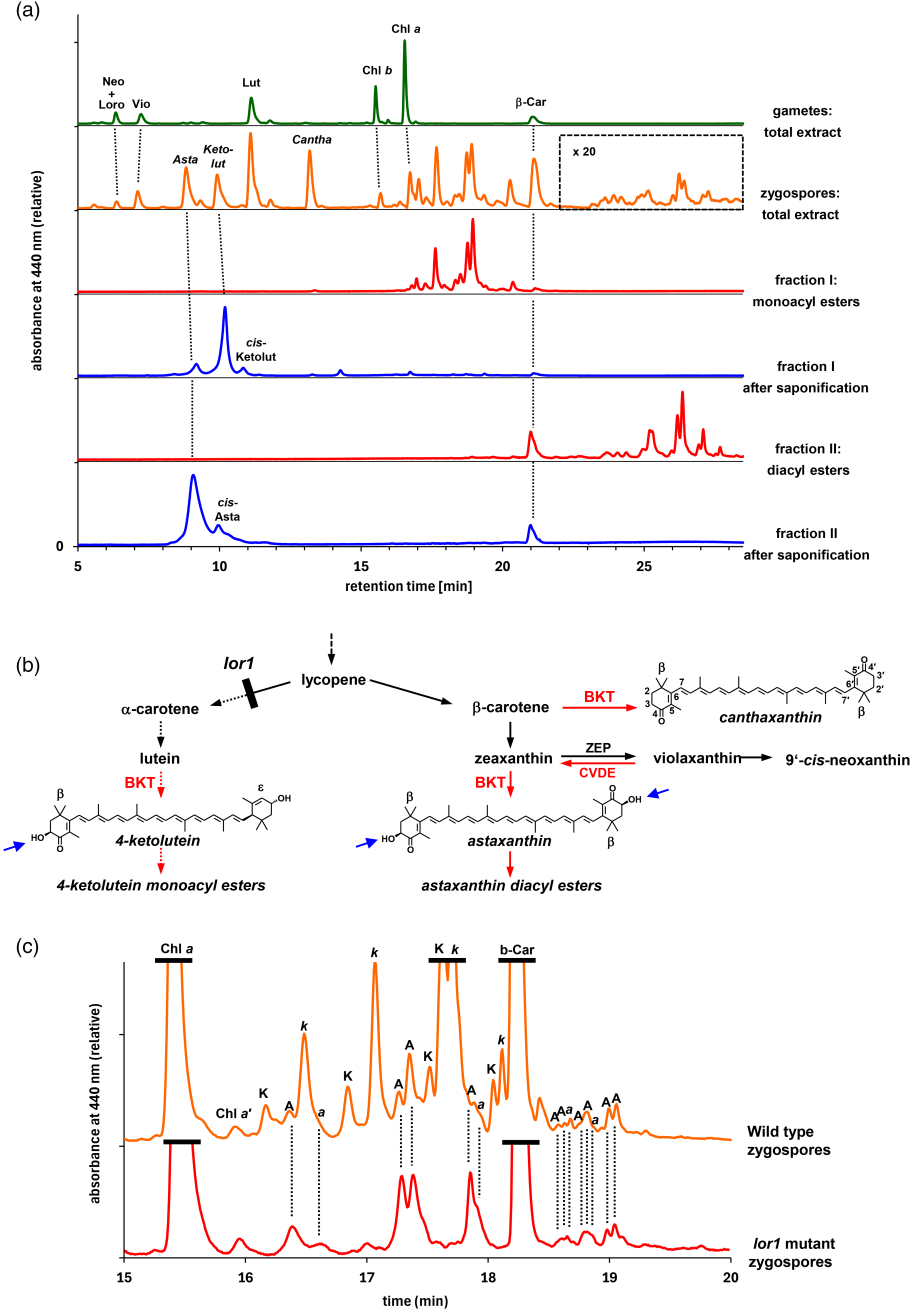
(a) HPLC analysis (Gradient I) of total pigment extracts from haploid vegetative cells (strain CC‐620) and diploid zygospores of *Chlamydomonas reinhardtii* after maturation in the dark for 3 months (in boxed part of chromatogram, y‐axis zoomed in 20‐fold), and saponification products from either fraction I containing the putative monoacyl esters or fraction II containing the putative diacyl esters of the ketocarotenoids from the zygospore extract. (b) Biosynthetic scheme illustrating the postulated zygospore‐specific pathway from photosynthetic carotenoids to ketocarotenoids indicated by arrows and enzyme names in red and names of zygospore‐specific pigments italicized. The pathway impaired in the *lor1* mutant is indicated by arrows with broken lines. In the chemical structures of the three major ketocarotenoids from the zygospores, blue arrows label the hydroxyl groups at C3 of the β‐ionone rings that can be esterified with fatty acids. Positions of the C‐atoms 2–7 and 2′–7′ are exemplified for canthaxanthin. (c) HPLC analysis (Gradient II) of ketocarotenoid acyl esters in *C reinhardtii* zygospores (22 days) resulting from homozygous crosses of either wild type (upper trace) or the *lor1*‐mutant (lower trace) which is deficient in α‐carotene and the xanthophylls derived thereof. Chromatograms were normalized to the β‐carotene peak. Dashed lines connect peaks of astaxanthin acyl esters present in both strains. A, *trans*‐astaxanthin acyl ester; *a*, 13‐*cis*‐astaxanthin acyl ester; Asta, astaxanthin; β, β‐ionone ring; BKT, β‐carotene ketolase; Cantha, canthaxanthin; Car, carotene; Chl, chlorophyll; Chl *a*′, Chlorophyll *a* epimer; CVDE, chlorophycean violaxanthin de‐epoxidase; ε, ε‐ionone ring; K, *trans*‐4‐ketolutein acyl ester; k, 13(′)*‐cis*‐4‐ketolutein acyl ester; Ketolut, 4‐ketolutein; Loro, loroxanthin; Lut, lutein; Neo, neoxanthin; Vio, violaxanthin; ZEP, zeaxanthin epoxidase.

For laboratory cultures of *H. pluvialis*, unfavorable environmental conditions inducing the accumulation of ketocarotenoids can be mimicked by nutrient starvation of batch cultures. During prolonged starvation of vegetative cells of *C. reinhardtii* in batch culture up to 3 months, we observed a reduction in chlorophylls and a relative increase of β‐carotene and lutein, but no additional pigments and in particular no ketocarotenoids were detectable. Nitrogen starvation induced gametogenesis of *C. reinhardtii*, but again no formation of ketocarotenoids was observed even after several weeks (Figure S1). When gametes of the two *C. reinhardtii* strains CC‐620 (mt^+^) and CC‐621 (mt^−^) were crossed, however, the resulting zygotes turned orange within a week of incubation in the dark at 22°C, and HPLC analyses of pigment extracts from matured zygospores revealed the presence of about 30 additional pigment peaks compared to vegetative cells (Figure 1a). A first survey of these peaks based on their on‐line UV/VIS‐spectra and retention times suggested the three most polar additional pigments in the HPLC chromatogram to be the ketocarotenoids astaxanthin, 4‐ketolutein [also known as fritschiellaxanthin (Buchecker et al., 1978)], and canthaxanthin (Figure 1).

The identification of astaxanthin and canthaxanthin in *C. reinhardtii* was facilitated by comparison with synthetic pigment standards. Both extracted and standard pigments showed identical retention times and on‐line spectra in our HPLC system. For both pigments from *C. reinhardtii*, the presence of keto groups at C‐atoms 4 and 4′ in the β‐ionone rings was supported by chemical reduction with NaBH_4_, which resulted in diagnostic changes of both the retention time and the UV/VIS‐spectra (Figure S2). In organic solvents or native lipid matrices, ketocarotenoids are reddish due to a bathochromic shift caused by the keto groups at C4(′) that are conjugated to the central polyene chromophore (Britton & Young, 1993); a chemical reduction of the keto groups abolishes their effect on the absorbance properties of the central chromophore (Eugster, 1995). After reduction with NaBH_4_, the tentative astaxanthin and canthaxanthin from *C. reinhardtii* displayed absorbance spectra very similar to zeaxanthin and β‐carotene indicating that all four pigments have the same chromophore (Table S1). Moreover, the HPLC analyses of partially reduced samples of both algal ketocarotenoids revealed the presence of an intermediate with only one of the two keto groups being reduced (see Figure S2), providing further support for their identification as 4,4′‐diketo‐carotenoids. Finally, mass spectrometric (MS) data of both pigments from *C. reinhardtii* were in accordance with the expected molecular weights of astaxanthin and canthaxanthin (Figure S3; Table S1).

For the pigment tentatively identified as 4‐ketolutein, no pigment standard was available. However, NaBH_4_‐mediated reduction led to a racemic product without detectable intermediates. The product displayed absorbance properties typical of lutein, indicating the presence of a single keto group at C4 of the β‐ionone ring that is conjugated with the extended π‐electron system comprising the 10 double bonds between C5 and C7′. The MS data further corroborated the proposed identity of the pigment, because in samples of the putative 4‐ketolutein and of lutein the major ion species corresponded to the [M‐18 + 1]^+^ molecule (Figure S3; Table S1); the abstraction of a water molecule in APCI‐MS has been described repeatedly as a diagnostic feature of ε‐ionone rings with a hydroxyl group at C3 (Aman et al., 2005; Dachtler et al., 2001; van Breemen et al., 1996). Taken together, these data strongly support the identification of the new ketocarotenoid from *C. reinhardtii* zygospores as 4‐ketolutein.

### The majority of ketocarotenoids in zygospores of *C. reinhardtii* are derived from α‐carotene

The comparison of pigment peaks with retention times between 16 and 21 min with HPLC chromatograms of pigment extracts from aplanospores of *H. pluvialis* reported in the literature (Breithaupt, 2004; Miao et al., 2006) suggested that these pigments were ketocarotenoid monoacyl esters. Likewise, those with retention times above 22 min were tentatively identified as diacyl esters of ketocarotenoids. Acyl esters of ketocarotenoids result from the formation of ester bonds between the hydroxyl groups at C3 of their β‐ionone rings (Figure 1b) with fatty acids of varying chain length. We then compared the on‐line UV/VIS‐spectra of the ketocarotenoid acyl esters with the spectra of the free chromophores astaxanthin and 4‐ketolutein and their *cis*‐isomers that we generated from the respective *trans*‐isomer pigment standards by thermal isomerization (Figure S4). This analysis permitted the provisional identification of the chromophore in each ester peak, because esterification of a ketocarotenoid with fatty acids at the C3‐ and C3′‐hydroxyl residues does not alter the chromophore and thus has no effect on its absorbance properties (Britton, 1995). The spectral data suggested most monoesters to be derived from 4‐ketolutein, whereas the minor amounts of diesters appeared to contain astaxanthin as the only chromophore. For further support of the chromophore assignment, we separately isolated the putative ketocarotenoid monoacyl esters eluting before β‐carotene and the putative diacyl esters eluting after β‐carotene and subjected them to saponification under nitrogen atmosphere. HPLC analyses of the saponified pigments showed that more than 90% of the monoacyl ester pool contained 4‐ketolutein as chromophore, whereas the diacyl ester pool contained exclusively astaxanthin as chromophore (Figure 1a). To obtain further proof of the chromophore nature of the ketocarotenoid acyl esters, we generated zygospores of the *lor1* mutant of *C. reinhardtii*, which fails to synthesize α‐carotene and its derivatives lutein and loroxanthin due to a defective lycopene‐ε‐cyclase (Figure 1b) (Anwaruzzaman et al., 2004). A HPLC analysis of the pigments from homozygous *lor1*‐zygospores revealed a significantly less complex composition of ketocarotenoid esters compared to the wild type (Figure 1c). In particular, all pigment peaks were absent that we had assigned as acyl esters of 4‐ketolutein based on their absorbance spectra, whereas the *lor1*‐zygospores had the same complement of tentative astaxanthin mono‐ and diacyl esters as the wild type.

### Ketocarotenoids in zygospores of *C. reinhardtii* are synthesized from photosynthetic carotenoids released during thylakoid breakdown and storage lipid accumulation

**Figure 2:**
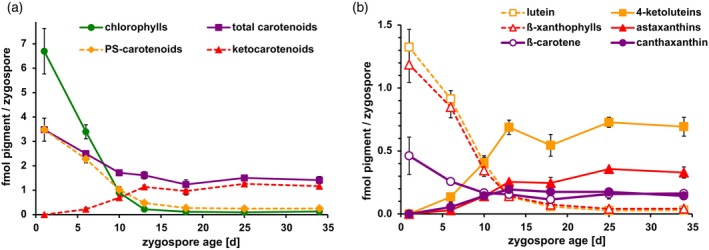
(a) Time course of decline in photosynthetic pigments and the parallel accumulation of ketocarotenoids in zygospores of *Chlamydomonas reinhardtii* during 33 days maturation in the dark (starting after 1 day in light) at 22°C. PS‐carotenoids, carotenoid species that were already present in vegetative (photosynthetic) cells (see Figure 1). (b) Time course of changes in the individual precursors and their ketocarotenoid products; 4‐ketoluteins and astaxanthins comprise the free ketocarotenoids and their respective acyl esters, while β‐xanthophylls include violaxanthin, antheraxanthin, and zeaxanthin (but not 9′‐*cis*‐neoxanthin that likely is no precursor of astaxanthin). Per time point, mean values of results from three zygote plates are shown (error bars denote standard deviations).

**Figure 3:**
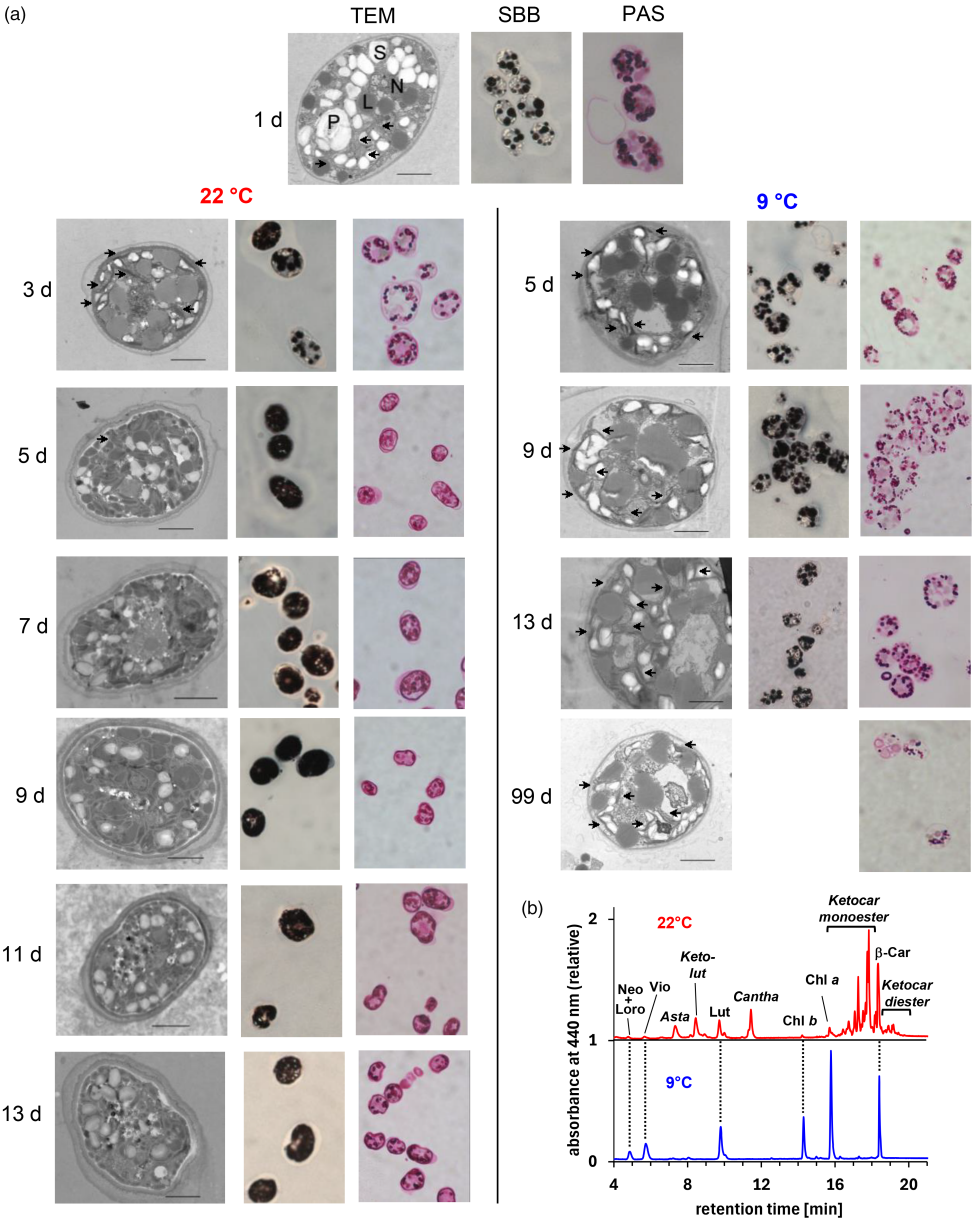
(a) Time series of TEM pictures showing ultrathin sections contrasted with uranyl acetate and lead salts and light microscopic pictures showing semithin sections of zygospores (numbers denote days after mating) either stained with Sudan Black B (SBB) to detect lipid accumulation or with Periodic acid Schiff (PAS) reagent staining polysaccharides with unsubstituted glycol groups. After 1 day in light, zygotes were dark‐incubated for up to 12 (98) days at either 22 or 9°C. Labels denote: L, lipids; N, nucleus; P, pyrenoid; S, starch; arrows, thylakoids; bars, 2 μm. (b) HPLC chromatograms (Gradient II) of pigment extracts from zygospores (99 days) after incubation either at 22 (upper red trace) or at 9°C (lower blue trace). Pigment abbreviations are as in Figure 1.

In *H. pluvialis*, the formation of aplanospores is accompanied by a massive increase in the cellular carotenoid content and an only moderate decrease in chlorophylls (Fábregas et al., 2003; Lemoine & Schoefs, 2010; Zlotnik et al., 1993). Dark‐incubated zygotes of *C. reinhardtii*, however, showed a strong decline in the content of chlorophylls and photosynthetic carotenoids within a few days after mating (Figure 2a). After 2–3 weeks, the chlorophyll content of the zygospores had decreased to 2% of the initial value and that of the ‘vegetative’ carotenoids to about 8%. The total carotenoid content, however, was still about 40% of the initial value due to the formation of ketocarotenoids, which accounted for almost 80% of cellular carotenoids in 18 days old zygospores (Figure 2a). The decline in total carotenoids indicated that the ketocarotenoids were derived from the photosynthetic carotenoids already present in the gametes. In line with this suggestion, the cellular increases of canthaxanthin, 4‐ketolutein plus its acyl esters, and astaxanthin plus its acyl esters were at all times lower than the corresponding decreases of their biosynthetic precursors lutein, β‐carotene, and the sum of violaxanthin, antheraxanthin and zeaxanthin, respectively (Figure 2b). As further support, and in line with earlier observations (Cavalier‐Smith, 1976), transmission electron microscopy of zygospores during a maturation period of 12 days in the dark (Figure 3a) showed that the overall decrease of photosynthetic pigments and the accumulation of ketocarotenoids (Figure S5) were accompanied by a strong reduction in thylakoid membranes and a massive accumulation of storage lipids throughout the cells. This was confirmed by *in situ*‐lipid staining with Sudan Black B of semithin sections from zygospores (Figure 3a) and by analysis of the lipid composition in lipophilic extracts from vegetative cells and mature zygospores by semiquantitative TLC (Figure S6); the latter contained mainly neutral triacylglycerols (TAG) and significantly less polar lipids than vegetative cells.

Notably, when zygospores were dark‐incubated at 9°C instead of 22°C, their maturation apparently stalled in an early stage—the cellular remodeling was less pronounced. In these zygospores, the neutral lipid droplets occupied a smaller volume, and patches of thylakoid membranes were detectable even after 3 months incubation (Figure 3a). Moreover, zygospores at 9°C did not establish the multilayered cell wall typical for mature zygospores as was apparent from the TEM images and confirmed by staining of the cell wall polysaccharides using the Phenolic acid Schiff (PAS) reaction (Figure 3a). In agreement with the postulated origin of zygospore ketocarotenoids from photosynthetic pigments, the zygospores at 9°C containing residual thylakoid membranes accumulated only minor amounts of ketocarotenoids and essentially retained all photosynthetic pigments (Figure 3b).

On the other hand, when zygospores developing at 22°C were incubated with norflurazon, a potent inhibitor of carotenoid biosynthesis, the accumulation of ketocarotenoids was not significantly different from non‐inhibited controls (Figure S7). After 21 days of dark incubation, the zygospores from the control plates showed the same cellular changes of chlorophylls, photosynthetic carotenoids and ketocarotenoids (Figure S7a,b) as observed before (Figure 2a). Application of norflurazon resulted in the accumulation of only small amounts of phytoene with most of the *de novo* synthesis already occurring during the 24 h light incubation period following zygote formation. This observation indicates (i) the successful inhibition of phytoene desaturase and (ii) a negligible activity of the carotenoid biosynthesis pathway during zygospore maturation. Between norflurazon concentrations of 10 and 25 μmol L^−1^, no significant difference in phytoene accumulation was detectable suggesting that a norflurazon concentration of 10 μmoL L^−1^ was sufficient for complete inhibition of the phytoene desaturase. The treatment with norflurazon did not result in a marked difference of ketocarotenoid contents per zygospore, whereas the concentrations of chlorophylls and photosynthetic carotenoids were noticeably higher than in untreated zygospores. However, this was also the case for zygospores on plates with only ethanol added, suggesting that the slightly reduced degradation of chlorophylls and photosynthetic carotenoids was caused by the addition of ethanol and not by norflurazon. The pigment content of zygospores after 35 days of maturation in the dark (Figure S7c) showed only minor differences compared to 21 days.

### The BKT gene from *C. reinhardtii* encodes a ketolase with broad substrate specificity and shows increased transcription in zygospores

**Figure 4:**
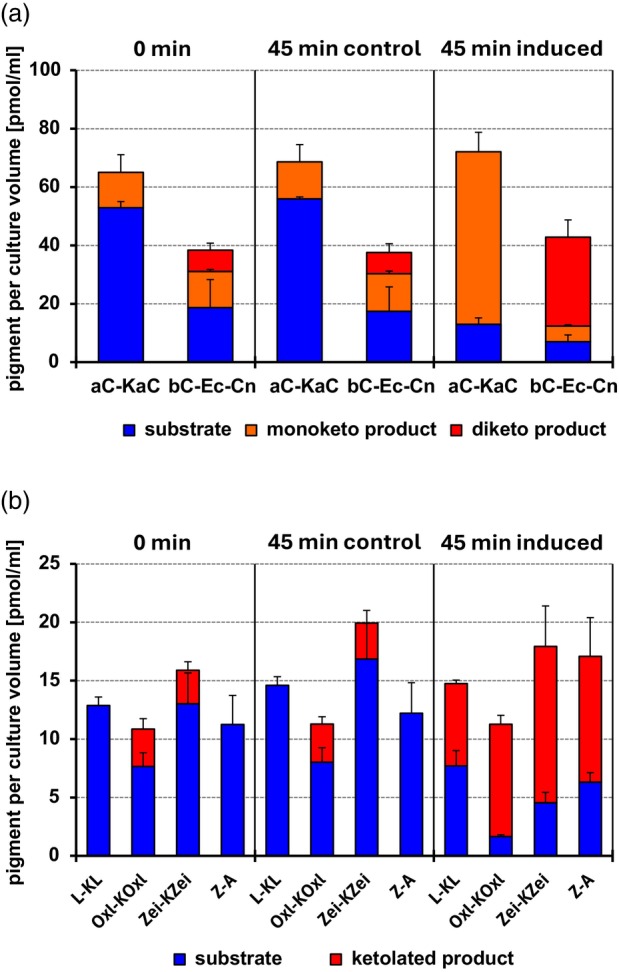
Activity of BKT from *Chlamydomonas reinhardtii* after heterologous expression in carotenogenic *Escherichia coli* strains supplying either (a) α‐carotene (aC) and β‐carotene (bC) or (b) lutein (L), 3′‐oxolutein (Oxl), zeinoxanthin (Zei) and zeaxanthin (Z) as substrates. Carotenoid concentrations in bacterial suspensions are shown at the start of induction (0 min) and after 45 min of growth in the absence (45 min control) or presence (45 min induced) of 0.04% arabinose. Abbreviations of ketolated products are: A, astaxanthin; Cn, canthaxanthin; Ec, echinenone; KaC, 4‐keto‐α‐carotene; KL, 4‐ketolutein; KOxl, 4‐keto‐3′‐oxolutein; KZei, 4‐ketozeinoxanthin. Data are average values of 4 biological replicates with error bars denoting standard deviations.

The BKT encoded in the genome of *C. reinhardtii* is the most likely candidate for catalyzing the conversion of photosynthetic carotenoids into the ketocarotenoids we detected in mature zygospores. The recombinant enzyme already was shown to accept β‐carotene and zeaxanthin as substrates (Huang et al., 2012; Zhong et al., 2011). To probe the substrate specificity of the BKT from *C. reinhardtii* towards the β,ε‐carotenoids α‐carotene and lutein (3,3′‐dihydroxy‐β,ε‐carotene), we ligated a 1.3 kb cDNA fragment spanning the complete open reading frame of the BKT gene into the arabinose‐inducible expression vector pBAD‐TOPO®. The resulting plasmid pBAD‐CrBKT was introduced into *Escherichia coli* XL1‐Blue cells containing either (i) plasmid pACCAR25ΔcrtX, which encodes bacterial genes necessary for formation of zeaxanthin (Kajiwara et al., 1995), (ii) its derivative pACCAR25ΔcrtXZ containing all genes necessary for β‐carotene synthesis, (iii) pALPHA1 inducing the simultaneous accumulation of α‐ and β‐carotene (Blatt et al., 2015) or (iv) pLUTEIN1 (Figure S8), a derivative of pALPHA1, leading to accumulation of lutein and 3′‐oxolutein in *E. coli* (Figure S9). In line with previous reports, arabinose induction of pBAD‐CrBKT in the β‐carotene background led to rapid formation of the monoketo‐carotene echinenone and the diketo‐carotene canthaxanthin (Figure S9c,e), and expression of CrBKT in *E. coli* cells containing zeaxanthin (3,3′‐dihydroxy‐β,β‐carotene) yielded astaxanthin (Figure S9d,f). Notably, induction of BKT in bacteria harboring pALPHA yielded 4‐keto‐α‐carotene in addition to canthaxanthin (Figure 4a; Figure S9a), and expression in cells accumulating lutein (3,3′‐dihydroxy‐β,ε‐carotene), zeinoxanthin (3‐hydroxy‐β,ε‐carotene) and oxolutein (3‐hydroxy‐3′‐keto‐β,ε‐carotene) resulted in the formation of 4‐ketolutein and the putative 4‐keto derivatives of zeinoxanthin and oxolutein, respectively (Figure 4b; Figure S9b). Thus, the recombinant BKT from *C. reinhardtii* ketolated β‐ionone rings containing a hydroxyl group at C3, even if the carotenoid substrate contained an ε‐ionone ring at the opposite end.

Although we failed to detect ketocarotenoids in vegetative cells of *C. reinhardtii*, the only EST clone of BKT had been isolated from a cDNA library generated from vegetative cells (Lohr et al., 2005). In support of this finding, we were able to amplify a BKT transcript not only from cDNA that was prepared from 5‐day‐old zygospores, but also from cDNA made from vegetative cells, though the level of BKT transcripts was much higher in the zygospores (Figure S10).

### Zygospores of *Volvox* also accumulate ketocarotenoids and bona‐fide BKT genes are present in the genomes of other Chlamydomonadales not yet known to accumulate ketocarotenoids

**Figure 5:**
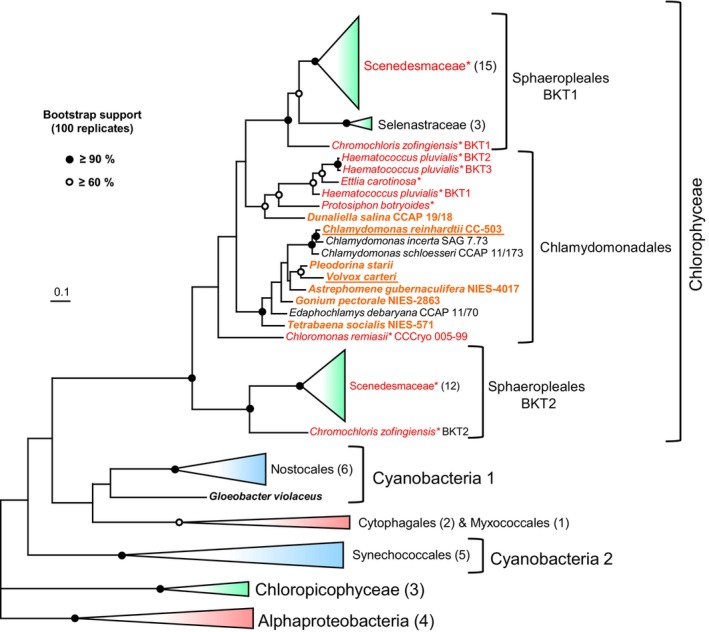
Phylogeny of BKT proteins from green algae and CrtW proteins from selected cyanobacteria and non‐photosynthetic bacteria. The maximum likelihood tree was inferred from an alignment of 70 protein sequences from 52 species and encompassing 245 amino acid positions and was rooted to a CrtW protein cluster from α‐proteobacteria. Nodes labeled with black dots had bootstrap support (100 replicates) ≥90%, white dots indicate support ≥60%. Green algal BKT proteins from species known to accumulate ketocarotenoids in the vegetative state are in red and labeled with an asterisk, those from species not known to accumulate ketocarotenoids but reported to generate reddish zygospores are in bold orange, and those for which we confirmed zygospore‐specific accumulation of ketocarotenoids are additionally underlined. See Table S2 for sequence accessions and Figure S11 for full tree with uncollapsed branches and bootstrap values.

A search for putative BKT genes in publicly available genome and transcriptome data of green algae (Chlorophyta) in Phycocosm (https://phycocosm.jgi.doe.gov/phycocosm/home) retrieved BKT sequences from algae of the order Sphaeropleales such as *Chromochloris zofingiensis* and many species of the family Scenedesmaceae, and from species of the Chlamydomonadales such as *H. pluvialis*, *Ettlia carotinosa* and *Protosiphon botryoides*, all of which are well‐known ketocarotenoid producers. In addition, we detected BKT candidates in the genomes of various Chlorophyta that have not yet been reported to accumulate ketocarotenoids, such as *V. carteri*, *D. salina*, *Gonium pectorale* and *Tetrabaena socialis* (Figure 5; Figures S11 and S12; Table S2), suggesting that in these algae the formation of ketocarotenoids may also be limited to the zygospores.

**Figure 6:**
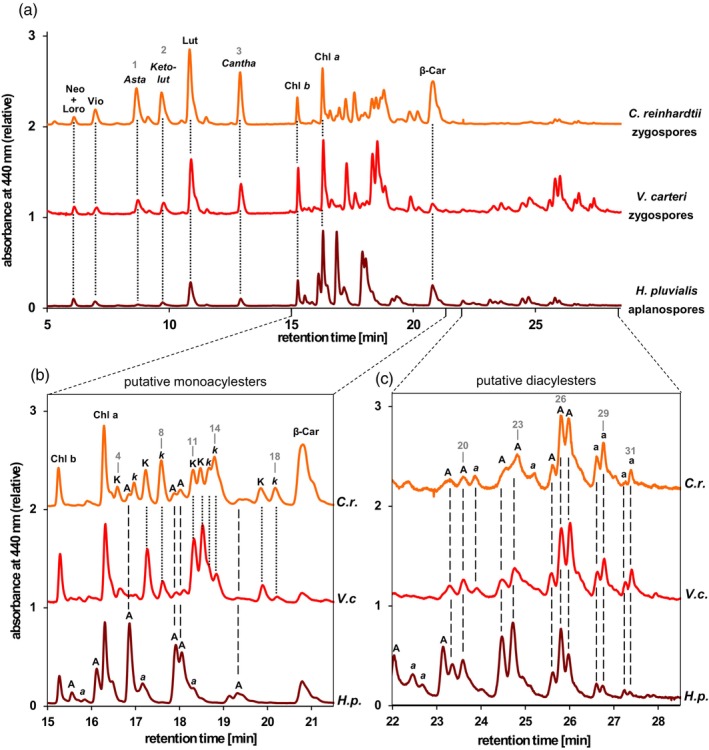
HPLC analysis (Gradient I) of total pigment extracts from diploid zygospores (33 days) of *Chlamydomonas reinhardtii* (*C.r*.), diploid zygospores (60 days) of *Volvox carteri* (*V.c*.), and haploid aplanospores (35 days) of *Haematococcus pluvialis* (*H.p*.). (a) Full chromatograms, (b) detail showing putative ketocarotenoid monoacyl esters, and (c) detail showing putative ketocarotenoid diacyl esters. Pigment abbreviations are: A, *trans*‐astaxanthin acyl esters; *a*, 13‐*cis*‐astaxanthin acyl esters; Asta, astaxanthin; Cantha, canthaxanthin; Car, carotene; Chl, chlorophyll; K, *trans*‐4‐ketolutein acyl esters; *k*, 13(′)‐*cis*‐4‐ketolutein acyl esters; Ketolut, 4‐ketolutein; Loro, loroxanthin; Lut, lutein; Neo, neoxanthin; Vio, violaxanthin.

In support of this hypothesis, an HPLC analysis of pigments extracted from a batch of about 150 000 deeply orange‐colored zygospores from *V. carteri* revealed the presence of various pigments with retention times and UV/VIS‐spectra identical to the ketocarotenoids from zygospores of *C. reinhardtii* (Figure 6). The zygospores of *V. carteri* showed a more balanced ratio of ketocarotenoids derived from α‐carotene versus those derived from β‐carotene as compared to zygospores from *C. reinhardtii* whose ketocarotenoids mainly derived from α‐carotene, and to *H. pluvialis* where essentially all ketocarotenoids were derived from β‐carotene (Figure 6).

## DISCUSSION

The green alga *C. reinhardtii* has been an outstanding model organism in photosynthesis research for more than half a century (Salomé & Merchant, 2019). Accordingly, the pigment composition of *C. reinhardtii* has been subject to numerous studies (reviewed in Lohr, 2023), and its carotenoid content has been analyzed under varying light conditions (Niyogi et al., 1997), during nutrient limitation (Czygan, 1968; Lohr et al., 2005; Wykoff et al., 1998) and throughout the vegetative cell cycle (Francis et al., 1975). However, to our knowledge, the occurrence of ketocarotenoids has not been reported previously. More than 40 years ago, Czygan (1968) mentioned that under nitrogen starvation some strains of the genus *Chlamydomonas* accumulated ketocarotenoids after zygote formation, but experimental data in support of this remark have not been published. We were prompted to investigate the carotenoid content of zygospores from *C. reinhardtii* and *V. carteri* by the discovery of BKT homologs in the publicly available genome sequences of these algae (Lohr et al., 2005 and this work), and found that in both species the formation of ketocarotenoids occurs in the diploid zygospores.

The presence of a BKT gene in the genomes of various other species of the Chlamydomonadales not yet known to synthesize ketocarotenoids (Figure 5) strongly suggests that in these algae the ketocarotenoid formation is also confined to the zygospore stage and that the ability to synthesize ketocarotenoids is widespread among green algae of the order Chlamydomonadales. In support of this hypothesis, reddish‐orange‐colored zygospores have already been reported for most of the species we found to possess a BKT gene, namely *Astrephomene gubernaculifera* (Brooks, 1966), *G. pectorale* (Hamaji et al., 2008), *D. salina* (Lerche, 1936), *Pleodorina starii* (Nozaki et al., 2006; Takahashi et al., 2021), and *T. socialis* (formerly *Gonium sociale*) (Nozaki, 1986). The other green algal BKT sequences we retrieved in our BLAST searches (Figures S11 and S12) were mainly from species of the order Sphaeropleales, whose vegetative stages are well‐known for their ability to accumulate ketocarotenoids during unfavorable growth conditions (Czygan, 1968; Goodwin, 1980). In conclusion, the detection of a putative BKT gene in a green alga can be regarded as a reliable predictor of the ability to synthesize ketocarotenoids in at least one of its life stages. On the other hand, the competence of algae to accumulate astaxanthin and other ketocarotenoids is not strictly linked to the presence of a BKT gene, as, for example, eustigmatophyte algae of the genus *Nannochloropsis* that possess secondary plastids of red algal origin also synthesize these pigments despite the lack of BKT candidates in their genomes (Cecchin et al., 2022; Gee et al., 2024).

A C‐terminally shortened version of the BKT from *C. reinhardtii* was already shown in carotenogenic bacteria to catalyze the ketolation of β‐carotene and zeaxanthin, that is, carotenoids with two β‐ionone rings (Huang et al., 2012; Zhong et al., 2011), but we are not aware of corresponding studies with the full‐length enzyme. In addition, clear evidence that the enzyme also accepts α‐carotene (β,ε‐carotene) and its hydroxylated derivative lutein as substrates has been lacking. Our functional expression of the full‐length BKT from *C. reinhardtii* in *E. coli* strains accumulating different carotenoid substrates (Figure 4; Figure S9) showed that the extended C‐terminus does not impair enzymatic activity in the bacteria. While we did not compare the activities of the full‐length BKT and the truncated version in our assay system, the rapid accumulation of ketocarotenoids in bacteria expressing the full‐length BKT argues against a negative impact of the extension on the catalytic activity. The observed substrate specificities agreed with previous results for the truncated version, showing that the enzyme efficiently ketolates both β‐carotene and zeaxanthin and discriminates only weakly between unsubstituted and C3‐hydroxylated β‐ionone rings (Huang et al., 2012; Zhong et al., 2011). In addition, we could show that the recombinant enzyme also ketolates the β‐ionone rings in α‐carotene, zeinoxanthin (3‐hydroxy‐α‐carotene) and lutein (3,3′‐hydroxy‐α‐carotene), indicating that the molecular structure of the end opposite to the ketolated β‐ionone ring is insignificant. Based on these observations, it appears unlikely that the biosynthesis of 4‐ketolutein in *C. reinhardtii* and *V. carteri* involves other ketolases in addition to the enzymes encoded by the single BKT genes which we found in the available genome data of these two algae.

Notably, the resting stages from *C. reinhardtii*, *V. carteri*, and *H. pluvialis* differed in the ratio of ketocarotenoids that were synthesized via α‐carotene or β‐carotene—that is, the ratio of 4‐ketolutein and its esters versus canthaxanthin plus astaxanthin and its esters (Figure 6). As discussed above, the substrate specificity of BKT is probably not a major determinant of this ratio. Instead, substrate availability is a likely key factor controlling the ratio of 4‐ketolutein versus astaxanthin formation in green algae. In support of this idea, heterologous expression of the BKT gene from *H. pluvialis* in seeds of *A. thaliana* (Stalberg et al., 2003) or in potato tubers (Morris et al., 2006), in which lutein is a major carotenoid, resulted in the accumulation of 4‐ketolutein and a concomitant decrease in lutein. Similarly, expression of the truncated version of the BKT gene from *C. reinhardtii* in the red alga *Cyanidioschyzon merolae* that accumulates β‐carotene, β‐cryptoxanthin and zeaxanthin as the sole carotenoids yielded the corresponding keto derivatives of these pigments (Seger et al., 2023). Studies in which the truncated BKT from *C. reinhardtii* was expressed in tobacco leaves (Huang et al., 2012), in lutein‐rich maize kernels (Farré et al., 2016) or in the alga itself (Perozeni et al., 2020; Tran & Kaldenhoff, 2020), however, found no 4‐ketolutein but only ketocarotenoids derived from β‐carotene. We can only speculate why no 4‐ketolutein was detected in these experiments, but it may have escaped detection in some of the HPLC analyses due to co‐elution with other pigments. Alternatively, 4‐ketolutein may have accumulated only in minor amounts due to low availability of its precursor lutein, which usually is tightly bound to the photosynthetic light harvesting complexes in green plastids (Bassi & Caffarri, 2000; Ruban et al., 1999).

In *H. pluvialis*, BKT has been detected in the plastid and the cytosol, but results from an *in vitro* assay indicated that only the cytosolic enzyme is active (Grünewald et al., 2001), and inhibitor studies support the idea that export of carotenoids from the plastid takes place on the level of β‐carotene (Grünewald & Hagen, 2001). *In vitro* assays with subcellular fractions suggested that both the ketolation and the subsequent formation of ketocarotenoid acyl esters take place at the endoplasmic reticulum (ER) and that ER‐associated diacylglycerol acyltransferases are involved in the latter step (Chen et al., 2015; Ma et al., 2022). Moreover, the formation of ketocarotenoids is closely linked to and depends on the accumulation of neutral lipids in the cytosol which constitute the solvent matrix for the lipophilic ketocarotenoid acyl esters (Boussiba, 2000; Chen et al., 2015; Schoefs et al., 2001; Zhekisheva et al., 2002, 2005). The BKT from *C. reinhardtii* contains a predicted chloroplast transit peptide at the N‐terminus (Lohr et al., 2005), and a fusion protein consisting of the N‐terminal 40 amino acids of BKT followed by yellow fluorescent protein (YFP) has been shown to be targeted into the chloroplast of *C. reinhardtii* (Perozeni et al., 2020). However, based on the striking similarities in the ultrastructure and lipid composition of aplanospores from *H. pluvialis* (Collins et al., 2011; Grünewald et al., 2001; Grünewald & Hagen, 2001; Santos & Mesquita, 1984; Wayama et al., 2013) and mature zygospores from *C. reinhardtii* (Cardador et al., 2025; Cavalier‐Smith, 1976; and Figure 3a; Figure S6), we postulate that in zygospores the ketolation of carotenoids by BKT and their subsequent acylation also take place at the ER and that the ketocarotenoid acyl esters accumulate in cytosolic TAG droplets. In support of this suggestion, the ketocarotenoids that accumulate inside the plastids of *C. reinhardtii* mutants ectopically expressing the truncated BKT are not esterified (Perozeni et al., 2020).

On the other hand, aplanospore formation in *H. pluvialis* is accompanied by a massive *de novo* synthesis of ketocarotenoids (Fábregas et al., 2003; Hoys et al., 2021; Recht et al., 2014; Zhao et al., 2020; Zlotnik et al., 1993), and the inhibition of carotenoid biosynthesis by norflurazon effectively prevents ketocarotenoid formation in this alga (Harker & Young, 1995). Conversely, the accumulation of ketocarotenoids in maturing zygospores of *C. reinhardtii* was not affected by norflurazon (Figure S7) but was accompanied by a massive loss of thylakoid membranes and an overall decrease in carotenoids. In addition, the cellular concentrations of the ketocarotenoids generated during zygospore maturation were always well below the concentrations of their presumptive precursor molecules present at the beginning of dark incubation (Figure 2b; Figure S5b) strongly supporting the conclusion that ketocarotenoid formation relies merely on the photosynthetic carotenoids provided by the degradation of the photosynthetic apparatus. According to this scenario (Figure 1b), canthaxanthin and 4‐ketolutein are synthesized by BKT from β‐carotene and lutein, respectively, whereas astaxanthin is derived from zeaxanthin that is generated from violaxanthin by the chlorophycean violaxanthin de‐epoxidase (CVDE) under increased excitation pressure on the photosynthetic apparatus (Li, Peers, et al., 2016). Similarly, the TAGs in the lipid bodies are probably at least in part synthesized from polar membrane lipids released during thylakoid breakdown (see Figure 3a and Figure S6, and the accompanying paper by Cardador et al. 2025).

Haploid cells of *C. reinhardtii* have also been shown to accumulate significant amounts of neutral lipids in cytosolic droplets under nitrogen starvation (Goodson et al., 2011; Moellering & Benning, 2010; Wang et al., 2009), but they do not develop into resting spores. We never detected ketocarotenoids in nitrogen‐starved haploid cells of *C. reinhardtii*, suggesting that some factors specific to the developmental program of resting spores (independent of ploidy) are important for ketocarotenoid formation. In this context, it is notable that incubation of zygospores from *C. reinhardtii* at 9°C not only affected the synthesis of ketocarotenoids and degradation of thylakoids but also the formation of the multilayered cell wall (Figure 3a). This observation agrees with previous results on a *C. reinhardtii* mutant defective in a type I polyketide synthase involved in the biosynthesis of the zygospore‐specific cell wall. Zygospores from this mutant were not only impaired in formation of the durable cell wall but also failed to degrade chlorophylls and to develop orange color (Heimerl et al., 2018), suggesting the process of cell wall reinforcement to be a key step for further cellular reorganization during zygospore maturation.

Furthermore, there is experimental evidence that BKT activity may be regulated both at the transcript and the protein level. EST data (Lohr et al., 2005), RNAseq analyses (Perozeni et al., 2020), qRT‐PCR assays (Chen et al., 2022) and our semiquantitative RT‐PCR results showed only a low basal transcription of the BKT gene in vegetative cells of *C. reinhardtii* while transcript levels were substantially higher in zygospores after 5 days dark incubation (Figure S10). On the protein level, the conspicuous C‐terminal extension of the BKT from *C. reinhardtii* apparently has no adverse effect on its catalytic activity in *E. coli* (Figure 4; Figure S9). However, *C. reinhardtii* mutants ectopically expressing the full‐length protein with a C‐terminal YFP tag showed a 30% lower accumulation of ketocarotenoids per cell than mutants expressing the truncated version fused to YFP, indicating that the C‐terminal extension has an effect on BKT activity in the algae (Perozeni et al., 2020). The extension may harbor a degradation signal that destabilizes the enzyme, similar to the N‐terminal extension in chlorophyllide *a* oxygenase (CAO) from *A. thaliana* and other vascular plants (Sakuraba et al., 2009). Notably, we found that the BKT proteins from the other Chlamydomonadales predicted to accumulate ketocarotenoids only in zygospores (with the exception of *D. salina*) also have substantially longer C‐termini than the ketolases from green algae that accumulate ketocarotenoids in the vegetative state (Figure S12). Contrary to the N‐terminal domain in plant CAO, the C‐terminal extensions in the algal BKT proteins share only low sequence identity without clear candidate regions for a conserved degron motif. Seven of the eight extensions, however, contain conspicuous poly‐alanine (poly‐Ala) repeats. Poly‐Ala repeats tend to self‐associate (Hayashi et al., 1999) and could promote the formation of BKT aggregates that may become prone to enhanced degradation via the proteasomal pathway in the cytosol or Clp proteases in the plastids (Isono et al., 2024). Such a mechanism could aid in suppressing a potential ketolase activity resulting from basal expression of BKT in vegetative cells.

Alternatively, the poly‐Ala repeats in the BKT proteins may associate with poly‐Ala repeats in other proteins, thereby playing a role either in a putative relocation of BKT from the dedifferentiating plastid to the ER or in the formation of multi‐enzyme metabolons for efficient biosynthesis of the ketocarotenoid esters. Notably, we found that the β‐carotene hydroxylases (CHYB) from the corresponding algae also contain short C‐terminal poly‐Ala repeats that are absent from the CHYB proteins of most other algae encoding a BKT (Figure S13). It is tempting to speculate that the poly‐Ala repeats in BKT and CHYB may facilitate the formation of CHYB‐BKT associations at the ER that promote the formation of astaxanthin from β‐carotene by substrate channeling. However, our observation that the ketocarotenoids in zygospores of *C. reinhardtii* are not synthesized *de novo* via carotenes but by recycling of photosynthetic xanthophylls—making the participation of CHYB at the site of their ketolation by BKT obsolete—renders this speculation less likely. It remains to be studied if the C‐terminal extension in the respective BKT proteins has indeed a functional relevance specific for ketocarotenoid accumulation in zygospores.

In the case of *H. pluvialis*, several potential functions of the astaxanthin esters in the cytosolic lipid globules have been considered, such as (i) acting as a sunscreen agent to protect the plastid and the nucleus from solar irradiation (Hagen et al., 1994; Wang et al., 2003; Yong & Lee, 1991); (ii) scavenging of reactive oxygen species (ROS) to protect cellular components like DNA, proteins and membranes (Gwak et al., 2014; Kobayashi et al., 1997), and (iii) function as an antioxidant to specifically protect storage lipids from peroxidation (Sun et al., 1998). In the *C. reinhardtii* mutants ectopically expressing the optimized BKT, on the other hand, the ketocarotenoids were not esterified and accumulated in the plastid, mainly as free pigments in the thylakoid membranes (Perozeni et al., 2020). Here, the ketocarotenoids appeared to act as both light filters and ROS scavengers, leading to lower photoinhibition and increased biomass productivity in high light (Cazzaniga et al., 2022). In the mature zygospores from *C. reinhardtii*, however, the thylakoids and photosynthetic pigments are strongly depleted and the bulk of ketocarotenoids are esterified and reside in the storage lipids that constitute a major portion of the dormant zygotes. For these ketocarotenoids, we propose a major role as powerful antioxidants that prevent lipid peroxidation and protect the energy reserves necessary for successful germination of the zygospores under favorable environmental conditions.

### *C. reinhardtii* as a new model for studying the metabolism of ketocarotenoids in green algae

Despite numerous studies, many questions remain about the formation and significance of ketocarotenoids in the cytosolic storage lipids of green algae. The mechanisms of export from the plastid of both the ketocarotenoid precursors and the fatty acids necessary for storage lipid formation are unknown (Benning, 2009; Chen et al., 2015; Grünewald et al., 2001; Ota et al., 2018), as are the route and mechanism of targeting of the BKT to the cytosolic lipid globules (Grünewald et al., 2001). Using *H. pluvialis*, the investigation of these questions is yet hampered by a limited molecular toolbox for this alga. The finding that *C. reinhardtii* is capable of ketocarotenoid synthesis paves the way for novel approaches to study green algal ketocarotenoid metabolism by exploiting the highly curated genomic and transcriptomic sequence data (Craig et al., 2023; Merchant et al., 2007) and versatile molecular genetic tools that have been developed for this long‐standing model organism (Crozet et al., 2018). Particular strengths of the model system *C. reinhardtii* are the availability of a number of pigment mutants which accumulate different amounts of the potential BKT substrates (Lohr, 2023) and of an extensive library of indexed mutants (Li et al., 2019; Li, Zhang, et al., 2016). Our exemplary experiment with the *lor1* mutant illustrates the power of *C. reinhardtii* as a model for studying green algal ketocarotenoid metabolism. The pigment mutants can now be exploited to investigate the influence of substrate availability on the ketocarotenoid composition and its consequences for the viability of the zygospores.

Our observations shed light on a biological facet of a largely unexplored resting stage that is critical for the survival and propagation of *C. reinhardtii* and many other green algae. The importance of this cell type contrasts with our current limited knowledge about the cytological and metabolic processes during zygospore differentiation. Studying the maturation and germination of green algal zygospores on the molecular level is still a challenge because of their highly resistant cell walls, which complicate the efficient extraction of cellular compounds like RNA or proteins. The anticipated results of such molecular studies, however, are likely to compensate for the efforts necessary to overcome current problems of sample preparation.

## EXPERIMENTAL PROCEDURES

### Algal strains, culture conditions, and generation of zygospores

*C. reinhardtii* strains CC‐620 (mt^+^), CC‐621 (mt^−^), *lor1* mt^+^ (ASX1‐1b), and *lor1* mt^−^ (ASX2‐11d) were grown as 200 mL‐batch cultures in Tris acetate phosphate (TAP) medium (Harris, 2009; Hui et al., 2023) at 22°C in an 8 h dark/16 h light (60 μmol photons m^−2^ sec^−1^) cycle and constant shaking at 120 rpm. Gametogenesis, crossing and zygote maturation were performed according to standard protocols (Harris, 2009). To induce gametogenesis, cells of opposite mating types were grown for 4–5 days, then pelleted by centrifugation for 10 min (2000×*g*) at 4°C, re‐suspended in TAP‐N (without NH_4_Cl) medium and incubated for 24 h at 60 μmol photons m^−2^ sec^−1^. The resulting gametes were pelleted by centrifugation (10 min, 2000×*g*, 4°C) and re‐suspended in TAP‐N to a final concentration of 1  ×10^8^ cells mL^−1^. Cell counts were done with a Zeiss Axioskop light microscope using disposable hemocytometers (C‐Chip Neubauer Improved; Peqlab); A minimum of two aliquots and 200 cells were counted per sample. For mating, equal volumes of both strains were mixed and incubated for 2–4 h at 60 μmol photons m^−2^ sec^−1^ and 22°C. Zygotes were plated on TAP‐N plates with 3% agar. Plates were incubated for 24 h at 60 μmol photons m^−2^ sec^−1^ and 22°C, then wrapped in aluminum foil and kept in the dark at 22°C until harvesting. To follow the time course of ketocarotenoid accumulation in the zygospores of *C. reinhardtii*, a larger batch of freshly mixed gametes was spread onto several TAP‐N plates that were harvested and analyzed in triplicate at different time points during maturation. For harvesting, zygospore‐containing agar plates were washed with sterile TAP‐N medium to remove unpaired gametes. The zygospores sticking to the agar surface were scraped from the plates with a rubber spatula and washed with TAP‐N. Residual agar was removed by consecutive passage of the zygospore suspension through finished‐cotton filters with 400 and 125 μm mesh size (Carl Roth, Germany).

The female *V. carteri* wild‐type strain “Isanuma *Volvox 6*” and the male wild‐type strain “Isanuma *Volvox 5*” were grown as 1 L‐cultures in *Volvox* medium at 28°C in an 8 h dark/16 h light (180 μmol photons m^−2^ sec^−1^) cycle (Starr & Jaenicke, 1974). For generation of zygospores, a population of vegetatively grown males was induced to produce and release the sex‐inducer using a heat shock (Kirk & Kirk, 1986). The fluid of this culture was sterile‐filtered and 7 mL of the filtrate were added to each 1‐L culture of vegetatively grown males or females at the developmental stage shortly before hatching of juveniles to induce sexual development. Ten synchronous 1‐L cultures of sexual, egg‐bearing females were mixed with ten synchronous 1‐L cultures of sexual males containing sperm‐packets and the resulting 2‐L cultures incubated for 10–20 days under standard conditions. Then, the maturing zygospores were separated from vegetative cell aggregates and incubated for another 3 weeks. For harvesting, the 10 mating batches of 2 L each were filtered through a 100 μm nylon mesh and a 40 μm nylon mesh resulting in the filtrate containing only zygotes, free reproductive cells, detached somatic cells and small cell sheets. The zygotes in the filtrate were enriched on a 10 μm nylon mesh, transferred to a petri dish with nitrogen‐free (liquid) *Volvox* medium, and incubated in the dark at 20°C for another 3 weeks. During this incubation, the zygotes were washed on the 10 μm nylon mesh with fresh nitrogen‐free medium once a day in order to remove all remaining cell debris. This procedure resulted in ~150 000 zygotes.

*Haematococcus pluvialis* strain SAG 192.80 was grown as 200 mL‐batch culture in Desmidiacean Medium (Schlösser, 1994) at 22°C without shaking in an 8 h dark/16 h light (40 μmol photons m^−2^ sec^−1^) cycle. Within 2 months, green cells turned into deep red hypnospores due to nutrient depletion.

### Pigment extraction, saponification, and HPLC analysis

General precautions for work with pigments were taken and standard methods for purification of pigments applied (Schiedt & Liaaen Jensen, 1995). For pigment analysis of algae, vegetative cells or zygospores were collected on GF 6 glass fiber filters (Whatman) under mild suction. Zygospores were washed two times with methanol to remove pigments from contaminating gametes (the cell wall of mature zygospores resisted extraction by methanol). Pigments from vegetative cells were extracted with methanol/ethyl acetate/water (8:1.2:1) buffered with 75 mmol L^−1^ ammonium acetate (ExMed I) and pigments from zygospores were extracted with methanol/dichloromethane (5:3; ExMed II); quantitative extraction of pigments by these solvent mixtures had been verified by systematic solvent tests. After addition of extraction medium, filter samples were ground in a Braun MSK cell homogenizer and cell debris was removed by centrifugation (15 000×*g*, 2 min) followed by HPLC analysis (Wilhelm et al., 1995; Lohr & Wilhelm, 1999). For extraction of pigments from carotenogenic *E. coli* cultures, 4 mL of a cell suspension were spun down at 6000×*g* for 10 min and the pellet was extracted by adding 30 μL ethyl acetate and 30 μL ExMed I followed by vortexing and ultrasonic treatment. After addition of another 190 μL ExMed I and centrifugation at 15 000×*g* for 3 min, the supernatant was diluted with 20% water (v/v) and subjected to HPLC analysis immediately after dilution.

Pigment extracts from zygospores were separated into mono‐ and diester fractions by loading 1.5 mL extract with a syringe onto three tandem Sep‐Pak® Plus tC18 cartridges (Waters WAT036800). The cartridges were washed by serial application of 1 mL water, 2 mL methanol/water (1:6) and 1 mL methanol. Then, pigments were eluted and collected in ten 1‐mL fractions by successive application of 3.5 mL methanol, 3 mL methanol/dichloromethane (9:1), 2 mL methanol/dichloromethane (6:1), and 1.5 mL dichloromethane. Fractions 5–7 containing the monoacyl esters were combined to yield “fraction I” in Figure 1(a), while fraction 10 contained the diacyl esters and represented “fraction II” in Figure 1(a).

For saponification, 1 mL of the respective fraction was diluted with 4 mL methanol and 1 mL dichloromethane, transferred to a 30 mL glass vessel, and sealed with a rubber stopper with two in‐/outlets and pierced by a syringe needle, through which nitrogen was bubbled gently for 10 min. Then, 1 mL of 0.2% (w/v) methanolic NaOH was added and the mixture incubated for 16 h in the dark at room temperature under continuous slow (<10 bubbles min^−1^) flushing with nitrogen. The saponification was terminated by thoroughly mixing the sample with 8 mL diethyl ether followed by stepwise addition of 5 M aqueous NaCl solution until phase separation occurred. The ether phase was collected and the remaining aqueous phase washed again with fresh ether. The two ether phases were combined and washed with water and aqueous NaCl solution until the pigmented ether phase became transparent. Then, the ether phase was collected in a clean glass vial and the ether evaporated to dryness by flushing with water‐free nitrogen gas. For HPLC analysis, the dried pigments were redissolved in ExMed I and diluted with 20% water (v/v) immediately before injection.

HPLC was done on a Waters Alliance 2795 Separation Module with a Waters 2996 photodiode‐array detector; data were collected and analyzed using Waters Empower software. Pigments were separated on a Chromolith® Performance RP‐18e 100‐4.6 mm column equipped with a Chromolith® RP‐18e 10‐4.6 mm guard cartridge (Merck) operated at 20°C by ternary gradients using either (i) Gradient I with a flow rate of 1 mL min^−1^ comprising linear changes from 70% eluent A (85% methanol buffered with 0.075 M ammonium acetate) and 30% eluent B (90% acetonitrile) at 0 min to 100% B at 4 min, to 80% B and 20% C (ethylacetate) at 10 min, to 60% B and 40% C at 13 min, 45% B and 55% C at 22 min, 30% B and 70% C at 25 min, which was kept until the end of the run after 30 min, or (ii) Gradient II with a flow rate of 1.5 mL min^−1^ comprising linear changes from 70% eluent A and 30% eluent B at 0 min to 100% B at 4 min, to 80% B and 20% C at 10 min, to 60% B and 40% C at 15 min, and to 30% B and 70% C at 17 min, which was kept until the end of the run at 23 min, or (iii) Gradient III with a flow rate of 1 mL min^−1^ and linear changes from 70% A and 30% B at 0 min to 100% B at 2 min, to 60% B and 40% C at 7 min, to 30% B and 70% C at 15 min, which was kept until the end of the run after 19 min. During each run, on‐line spectra were recorded between 270 and 700 nm with 1.2 nm resolution and a sampling rate of 2 sec^−1^. The xanthophylls loroxanthin and neoxanthin were not separated by the three HPLC systems described. Some pigment extracts were analyzed using a ProntoSIL 200‐5 C30‐column (Bischoff Analysentechnik, Leonberg, Germany) operated at 28°C with Gradient IV, using a flow rate of 1.3 mL min^−1^ and linear changes from 10% eluent A and 90% eluent B at 0 min to 100% B at 0.75 min, to 60% B and 40% C at 2.5 min, 50% B and 50% C at 8 min, 10% B and 90% C at 23 min, 10% B and 90% C at 26 min, and 100% C at 26.5 min until the end of the run at 32 min. Using this gradient, we found the loroxanthin level of vegetative cells of *C. reinhardtii* strains CC620 and CC621 grown as specified to be below 5% of total carotenoids.

### Identification of ketocarotenoids

The identification of ketocarotenoids from *C. reinhardtii* and from the BKT assay in carotenogenic *E. coli* was based on comparison with reference pigments, diagnostic chemical derivatization reactions, and LC/MS analysis. The reference pigments used were synthetic astaxanthin (racemic mixture of three possible isomers at the C3 and C3′ hydroxyl groups) and canthaxanthin (both kindly donated by BASF) and natural astaxanthin and canthaxanthin from *H. pluvialis*. *Cis*‐isomers of astaxanthin and 4‐ketolutein were tentatively identified by on‐line absorbance spectra using the products of thermal isomerization of both pigments as reference; thermal isomerization of the all‐*trans*‐isomers was performed according to Marx et al. (2003). The presence of ketogroups in astaxanthin, canthaxanthin and 4‐ketolutein from *C. reinhardtii* was investigated by chromatographic separation of each pigment and subsequent reduction with NaBH_4_ according to Eugster (1995). For LC/MS analyses, astaxanthin, canthaxanthin, 4‐ketolutein and lutein were isolated from mature zygospores of *C. reinhardtii* and a pooled sample containing about 100 ng of each pigment in methanol/ethyl acetate/water (8:1.2:1) was subjected to LC–MS on a HPLC system consisting of a P 580 LPG pump and a UVD 340 S detector (Gynkotek/Dionex) coupled to a PE‐SCIEX QStarPulsar mass spectrometer (Applied Biosystems/PE Sciex) and operated with Chromeleon 6.4 software (Dionex). Pigments were separated using a ProntoSIL 200‐5 C30, 5.0 μm, 250 × 4.6 mm column equipped with a ProntoSIL 200‐5‐C30, 5.0 μm, 20 × 4.0 mm guard column (Bischoff Analysentechnik, Leonberg, Germany) operated at 20°C with a binary gradient (flow 0.9 mL min^−1^) of linear changes from 90% eluent A (92% methanol) and 10% eluent B (100% tert‐butyl methyl ether) to 35% A at 35 min kept until the end of the run at 52 min. Ionization of samples was achieved by atmospheric pressure chemical ionization (APCI) at 450°C and the total ion current was detected in the positive ion mode.

### Treatment of zygospores from *C. reinhardtii* with the inhibitor norflurazon

For the inhibitor experiments, a 10 mmol L^−1^ ethanolic stock solution of the phytoene desaturase inhibitor norflurazon (Sigma‐Aldrich) was spread onto Petri plates containing 20 mL TAP agar to yield inhibitor concentrations of 5, 10 or 25 μmol L^−1^ (final ethanol concentration between 0.05 and 0.25% v/v). To control plates, 50 μL of pure ethanol (final concentration of 0.25% v/v) were spread to check for potential adversary effects of the solvent on zygospore maturation. Untreated TAP plates were used as further control. Freshly prepared zygospores were spread on the five different plate types. The plates were incubated for 24 h at 60 μmol photons m^−2^ sec^−1^ and 22°C, then wrapped in aluminum foil and kept in the dark at 22°C. After 21 and 35 days of dark incubation, two plates from each of the five different treatments were harvested and separately analyzed for pigment contents of the zygospores.

### Lipid extraction and analysis

*C. reinhardtii* samples were processed like the samples for pigment extraction but using chloroform/methanol (2:1) as solvent. Total lipids were separated on 5‐cm × 7.5‐cm HPTLC Kieselgel 60 aluminum sheets (MERCK) by a two‐step chromatographic development and visualized as described in Grünewald et al. (2001). Polar lipid standards were obtained from Lipid Products (South Nutfield, Great Britain). The TAG standard was cold‐pressed sunflower oil (EDEKA Group, Germany).

### Functional expression of the BKT gene from *C. reinhardtii* in carotenogenic *E. coli* strains

Applying standard methods (Sambrook & Russell, 2001), a 1.3 kb cDNA fragment containing the complete ORF of the BKT gene from *C. reinhardtii* (GenBank AY860820) was cloned into the pBAD‐TOPO® expression vector (Invitrogen) in frame with the vector‐encoded C‐terminal poly‐histidine tag. The resulting plasmid pBAD‐CrBKT encoded the full‐length BKT (444 amino acids) extended by 14 amino acids (MGSGSGDDDDKLAL) at the N‐terminus and 28 amino acids (KGELEGKPIPNPLLGLDSTRTGHHHHHH) at the C‐terminus. For the purified plasmid, the insertion region was sequenced on both strands (GENterprise, Mainz, Germany) and found to be free of errors. Plasmid pACCAR25ΔcrtXZ encoding bacterial genes for formation of β‐carotene was generated from plasmid pACCAR25ΔcrtX (Kajiwara et al., 1995) by disrupting the CrtZ gene encoding β‐carotene hydroxylase; pACCAR25ΔcrtX was double‐digested with *Aat*II and *Nsi*I, sticky ends were filled by incubation with dNTPs and Phusion DNA‐Polymerase (New England Biolabs) and religated. Plasmid pLUTEIN1 was constructed by introducing the CrtZ gene encoding a bacterial carotene hydroxylase from plasmid pACCAR25ΔcrtX into plasmid pALPHA1 (Blatt et al., 2015). pACCAR25ΔcrtX and pALPHA1 were double‐digested with *Xba*I and *Nde*I, the resulting 2.5 kb fragment from pACCAR25ΔcrtX and the 9.3 kb fragment from pALPHA1 gel‐purified and ligated. The resulting plasmid pLUTEIN1 was transformed into *E. coli* TOP10 cells and found to cause the simultaneous accumulation of lutein, 3′‐oxolutein, zeinoxanthin and zeaxanthin. pBAD‐CrBKT was cotransformed into *E. coli* TOP10 together with either pACCAR25ΔcrtX, pACCAR25ΔcrtXZ, pALPHA1 or pLUTEIN1. Double transformants containing both plasmids were selected on LB agar supplemented with chloramphenicol (30 mg L^−1^) and ampicillin (100 mg L^−1^). Double transformants were grown in liquid LB medium under selection pressure at 28°C to an OD_600_ of 0.7 to 0.9, split in two aliquots and expression of BKT was induced in one of the aliquots by addition of 0.04% (w/v) arabinose.

### Preparation of cDNA and semiquantitative PCR of BKT transcripts

For cDNA preparation, mRNA was isolated from 10 mL of a suspension of vegetative cells (approx. 10^7^ cells per mL) of strain CC621 (5 days after inoculation), and from zygospores of crosses of CC620 × CC621 after 5 days of maturation on agar plates. Cells were pelleted by centrifugation (3000*g*, 5 min) and zygospores were harvested on GF6 filters as described above. Using the High Pure RNA Isolation Kit (Roche), 400 μL lysis buffer was added to vegetative cell pellets or zygospores on filters, and samples were transferred to FastPrep Lysing Matrix B Tubes (MP Biomedicals). Cells were disrupted by shaking in a Mini‐BeadBeater‐1 (BioSpec Products) applying 3 intervals of 10 sec at 3000 rpm with intermittent cooling of samples on ice. After addition of another 400 μL lysis buffer and brief centrifugation, the supernatant was transferred to a spin column and further processed according to the manufacturer's instructions. Total RNA was eluted in 50 μL of water and quantified using a BioPhotometer (Eppendorf), and its integrity was checked by agarose gel electrophoresis.

700 ng total RNA from vegetative cells or 230 ng total RNA from zygospores were reverse transcribed at 53°C using the Transcriptor High Fidelity cDNA‐System (Roche) following the manufacturer's instructions. Transcripts of BKT and of chlorophyllide a oxygenase (CAO) were detected by semiquantitative RT‐PCR using a T Gradient‐Thermocycler (Biometra) and the GC‐RICH PCR System (Roche) with intron‐spanning primers CrBKT‐end‐a2p (TACCACTTCGACCTGCACTG) and CrBKT‐3UTR‐e1m (GCCACGTGTGCCATGTTA) for BKT (expected product length of 688 bp; contaminant genomic DNA 987 bp) or CrCAO‐end‐a2p (AGACGCTGCCGGACTTC) and CrCAO‐3UTR‐e1m (CCGCTCTATCCTCCACC) for CAO (expected product length of 765 bp; contaminant genomic DNA 927 bp). 2 μL of product from first‐strand synthesis was added to the PCR reactions; the control reactions contained 2 μL of ultrapure water, instead. The optimized PCR‐conditions were as follows: initial heating to 95°C for 3 min, followed by 38 cycles of melting at 95°C for 30 sec, annealing at 59°C (BKT) or 61°C (CAO) for 30 sec, and elongation at 72°C for 90 sec; the final elongation time was 7 min. PCR products were separated on a 1% agarose gel using TAE buffer, stained with ethidium bromide, and visualized on a Gel Doc 1000‐System using Molecular Analyst 1.4 software (Bio‐Rad).

### Sequence data mining and phylogenetic analyses

The amino acid sequence of the BKT from *C. reinhardtii* (GenBank accession AAX54908) was used for BLAST (Altschul et al., 1997) searches in (i) the PhycoCosm database at https://phycocosm.jgi.doe.gov/phycocosm/home (Grigoriev et al., 2021) and (ii) the protein sequence data of various cyanobacteria and proteobacteria in the GenBank microbial genomes to retrieve bacterial CrtW sequences used in previous phylogenetic analyses (Makino et al., 2008). The protein sequences were aligned using the online version of MAFFT v7.511 (Katoh et al., 2019) with default settings (Strategy ‘Auto’; Scoring matrix ‘BLOSUM62’; Gap opening penalty ‘1.53’; Offset value ‘0.0’; Guide tree ‘Default’; Mafft‐homologs off) followed by manual refinement and clipping of ambiguously aligned parts in BioEdit 5.0.9 (Hall, 1999). Based on the clipped alignment, the maximum likelihood (ML) tree was inferred using PThreads in RAXML 8.2.10 (Stamatakis, 2014) and the Whelan and Goldman substitution model (Whelan & Goldman, 2001) with gamma rate distribution (option ‘‐m PROTGAMMAWAG’, four discrete rate categories). For branch support estimation, 100 bootstrap replicates were performed using the rapid bootstrap algorithm (Stamatakis et al., 2008) implemented in RAxML PThreads (option ‘‐f a’). The phylogenetic tree was exported from TreeView 1.6.6 (Page, 1996) and refined in PowerPoint (Microsoft Office).

### Fixation and embedding of cells for microscopy

Preparation of samples from vegetative cells and zygospores of *C. reinhardtii* for transmission electron microscopy was done according to van Winkle‐Swift and Rickoll (1997) with minor modifications. Cells were pelleted by microcentrifugation and embedded (at high cell density) in ultra‐low temperature gelling agarose (2.5% agarose in TAP‐N medium). The agar blocks were cut in smaller pieces, transferred to 2% glutaraldehyde (in TAP‐N) and fixed overnight at 4°C. Following repeated washing in 5 mmol L^−1^ Sörensen's phosphate buffer (pH 7.4, 3 × 30 min), the agar‐embedded cells were postfixed overnight at 4°C in 1% osmium tetroxide (in phosphate buffer). After washing in Sörensen's phosphate buffer (3 × 30 min), the agar blocks were dehydrated by 30, 50, 70, 90, 95, and 3 × 100% ethanol for 15 min respectively. The dehydrated agar blocks were subsequently infiltrated with 2:1, 1:1 and 1:2 mixtures of ethanol: Spurr's medium (Spurr, 1969) overnight at 4°C. Thereafter, the agar blocks were transferred 4 times to pure, fresh Spurr's medium over the next 2 days. Finally, the agar blocks were embedded in pure, fresh Spurr's medium in flat molds and cured at 65°C overnight.

### Transmission electron microscopy of ultrathin cell sections

Ultrathin sections (60 nm) of embedded cells were prepared with a Ultracut E ultramicrotome (Reichert‐Jung/Leica) equipped with a diamond knife (Diatome) and collected on formvar‐coated copper grids (75 mesh). Sections were successively contrasted with uranyl acetate (2% ethanolic solution) for 10 min followed by a triple lead stain according to Hanaichi et al. (1986) for 2 min, washed, air‐dried, and subjected to TEM analysis. Sections were viewed with a FEI Tecnai 12 BioTwin transmission electron microscope (FEI Europe, Eindhoven, The Netherlands) at 120 kV and imaged with a SCCD SIS MegaView III camera (Olympus Soft Imaging Solutions, Münster, Germany).

### Light microscopy of cell sections stained with Sudan Black B or by the PAS reaction

Semithin sections (1 μm) of embedded cells were also prepared with a Ultracut E ultramicrotome, using self‐made glass knives. To detect lipids, sections were stained with Sudan Black B (SBB, 0.3% in 70% ethanol) for 1 h at 60°C, followed by rinsings with 70% ethanol and distilled water. Lipids stain black, while cell walls and starch grains remain uncolored. Polysaccharides such as starch and cellulose were stained with the PAS (Periodic Acid Schiff) reaction. Following oxidation with periodic acid (5% aqueous solution) for 30 min at 50°C and repeated rinsings with distilled water, sections were stained with Schiff's reagent (Carl Roth, Karlsruhe) for 20 min at 50°C, also followed by repeated washings with distilled water. PAS‐positive structures stain purple‐magenta. Light microscopic images were taken using a Zeiss Axioskop trinocular light microscope (Carl Zeiss Microimaging) equipped with a Canon Powershot 640 digital camera. Differential interference contrast microscopic images were acquired on a Zeiss Axioplan DIC microscope (Carl Zeiss) equipped with a Moticam 2300 digital camera (Motic).

## AUTHOR CONTRIBUTIONS

ML designed research; SS, MB, VS, and ML performed research; AH contributed new reagents/material, SS, MB, VS and ML analyzed data; and ML wrote the manuscript. All authors reviewed the results and the manuscript.

## CONFLICT OF INTEREST STATEMENT

The authors declare no conflicts of interest.

## Supporting information


**Figure S1.** HPLC analysis of total pigment extracts from haploid gametes of the *Chlamydomonas reinhardtii* strains CC‐620 and CC‐621 after 33 days incubation in the dark at 22°C on TAP agar plates.
**Figure S2.** HPLC chromatograms and on‐line absorbance spectra of major peaks after diagnostic reduction by NaBH_4_ of putative ketocarotenoids isolated from zygospores of *Chlamydomonas reinhardtii* and of reference pigments canthaxanthin and astaxanthin.
**Figure S3.** Mass spectrometric identification of ketocarotenoids extracted from zygospores of *Chlamydomonas reinhardtii*.
**Figure S4.** HPLC on‐line absorbance spectra of different isomers of astaxanthin and 4‐ketolutein generated by thermal isomerization in toluene at 105°C for 15 min.
**Figure S5.** Time course of decline in photosynthetic pigments and the parallel accumulation of ketocarotenoids in samples from the zygospores of *Chlamydomonas reinhardtii* during 12 days maturation in the dark at 22°C.
**Figure S6.** TLC analysis of lipid classes in total lipid extracts from vegetative cells and from zygospores of *Chlamydomonas reinhardtii* matured at either 22 or 9°C for 98 days.
**Figure S7.** Ketocarotenoid accumulation in zygospores maturing on TAP plates treated with three different concentrations of the phytoene desaturase inhibitor norflurazon dissolved in ethanol versus zygospores on untreated plates and zygospores on plates containing 0.7% (v/v) ethanol.
**Figure S8.** Vector map of plasmid pLUTEIN1 that induces the accumulation of lutein, 3′‐oxolutein, zeinoxanthin and zeaxanthin in *Escherichia coli*.
**Figure S9.** Activity of BKT from *Chlamydomonas reinhardtii* after heterologous expression in *Escherichia coli* strains engineered to supply different carotenoids as substrates.
**Figure S10.** Result of reverse‐transcriptase PCR using total RNA prepared from 5 days old zygospores or from vegetative cells of *Chlamydomonas reinhardtii*.
**Figure S11.** Phylogeny of BKT proteins from green algae and CrtW proteins from selected cyanobacteria and other eubacteria.
**Figure S12.** Protein sequence alignment of BKT from algae and CrtW from selected cyanobacteria and other eubacteria.
**Figure S13.** Partial protein sequence alignment showing the C‐termini of CYHB enzymes from green algae for which the BKT sequences in Figure S12 were identified in publicly accessible databases.

**Table S1.** Identification of major free ketocarotenoids in pigment extracts from mature zygospores of *Chlamydomonas reinhardtii*.
**Table S2.** Accessions of BKT/CrtW sequences in alignment of Figure S12 (used to infer the ML tree in Figure 5 and Figure S11) and of CHYB in Figure S13.

## Data Availability

All study data mentioned in this article are included in the manuscript and the supplementary material. The sequence data used in this work are publicly available and their accession codes are supplied in the supplementary material.
