# Supplementary material for: Chlamydomonas reinhardtii, Volvox carteri and related green algae accumulate ketocarotenoids not in vegetative cells but in zygospores
Source: Plant J. 2025 Feb 9;121(3):e17261. doi: 10.1111/tpj.17261 (PMC11808291; doi:10.1111/tpj.17261)
Supplement: Supplementary file 1 — Figure S1. HPLC analysis of total pigment extracts from haploid gametes of the Chlamydomonas reinhardtii strains CC‐620 and CC‐621 after 33 days incubation in the dark at 22°C on TAP agar plates. Figure S2. HPLC chromatograms and on‐line absorbance spectra of major peaks after diagnostic reduction by NaBH4 of putative ketocarotenoids isolated from zygospores of Chlamydomonas reinhardtii and of reference pigments canthaxanthin and astaxanthin. Figure S3. Mass spectrometric identification of ketocarotenoids extracted from zygospores of Chlamydomonas reinhardtii. Figure S4. HPLC on‐line absorbance spectra of different isomers of astaxanthin and 4‐ketolutein generated by thermal isomerization in toluene at 105°C for 15 min. Figure S5. Time course of decline in photosynthetic pigments and the parallel accumulation of ketocarotenoids in samples from the zygospores of Chlamydomonas reinhardtii during 12 days maturation in the dark at 22°C. Figure S6. TLC analysis of lipid classes in total lipid extracts from vegetative cells and from zygospores of Chlamydomonas reinhardtii matured at either 22 or 9°C for 98 days. Figure S7. Ketocarotenoid accumulation in zygospores maturing on TAP plates treated with three different concentrations of the phytoene desaturase inhibitor norflurazon dissolved in ethanol versus zygospores on untreated plates and zygospores on plates containing 0.7% (v/v) ethanol. Figure S8. Vector map of plasmid pLUTEIN1 that induces the accumulation of lutein, 3′‐oxolutein, zeinoxanthin and zeaxanthin in Escherichia coli. Figure S9. Activity of BKT from Chlamydomonas reinhardtii after heterologous expression in Escherichia coli strains engineered to supply different carotenoids as substrates. Figure S10. Result of reverse‐transcriptase PCR using total RNA prepared from 5 days old zygospores or from vegetative cells of Chlamydomonas reinhardtii. Figure S11. Phylogeny of BKT proteins from green algae and CrtW proteins from selected cyanobacteria and other eubact [file TPJ-121-0-s001.pdf]

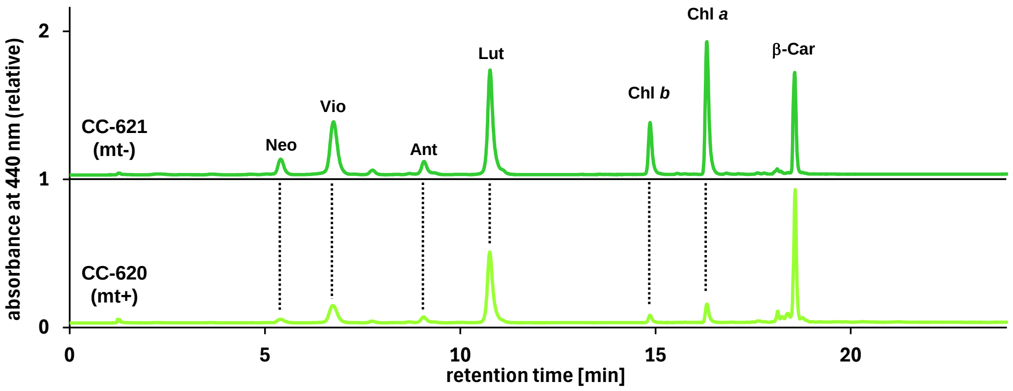

Fig. S1: HPLC analysis (Gradient II) of total pigment extracts from haploid gametes of the *C. reinhardtii* strains CC-620 and CC-621 after 33 d incubation in the dark at 22°C on TAP agar plates. Pigment abbreviations are: Ant, antheraxanthin; Car: carotene; Chl, chlorophyll; Lut: lutein; Neo: neoxanthin; Vio: violaxanthin.

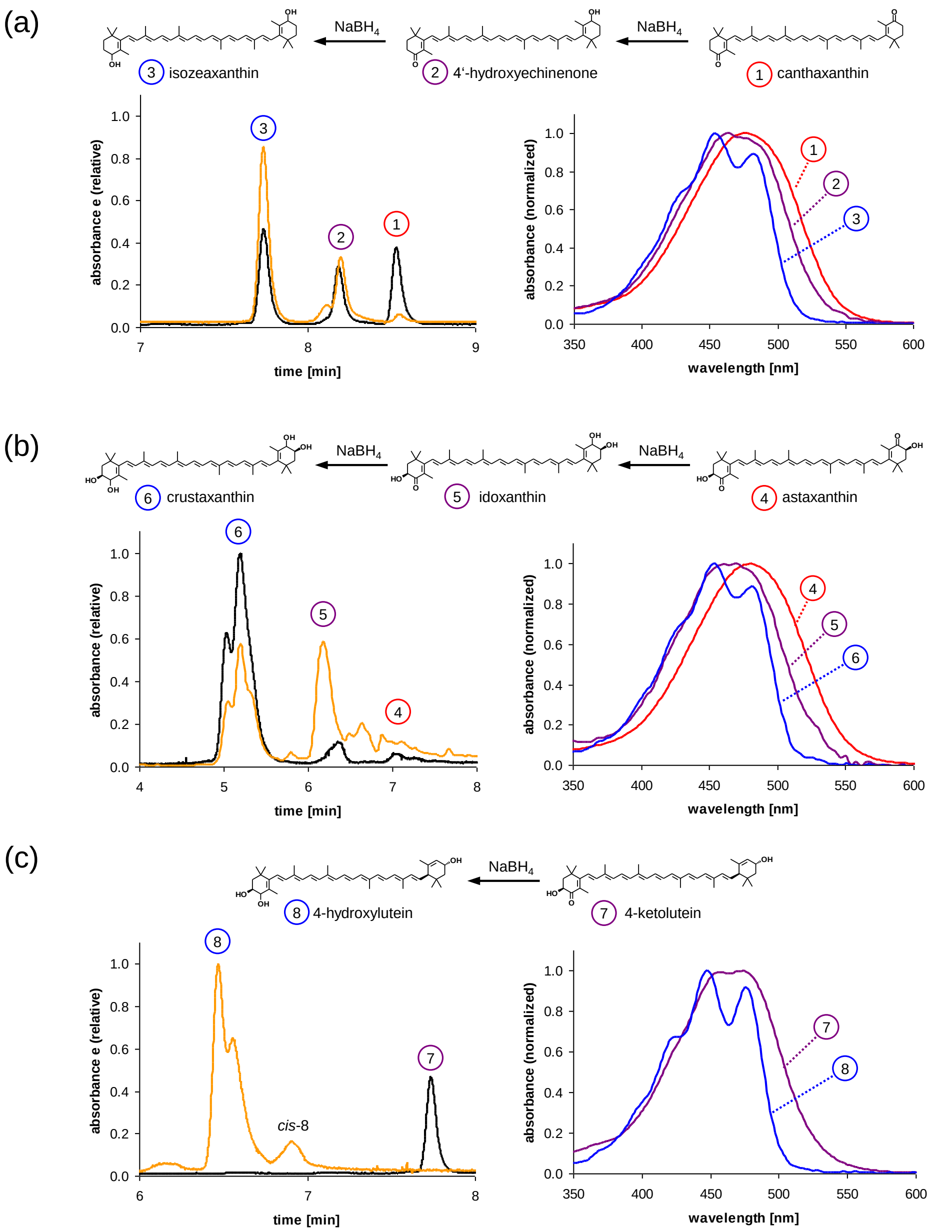

Fig. S2: HPLC chromatograms (Gradient III) and on-line absorbance spectra of major peaks after diagnostic reduction by $NaBH_4$ of putative ketocarotenoids isolated from zygospores of *C. reinhardtii* (orange chromatograms) and of reference pigments canthaxanthin and astaxanthin (black chromatograms). Note that reduction of a keto group by $NaBH_4$ yields a racemic mixture of diastereomers with the hydroxyl group in either R- or S-configuration that elute in short succession. **(a)** Reduction of the two keto groups in canthaxanthin (peak 1) yielded isozeaxanthin (β,β-carotene-4,4'-diol; peak 3) via 4'-hydroxyechinenone (peak 2); **(b)** reduction of the two keto groups in astaxanthin (peak 4) yielded crustaxanthin (β,β-carotene-3,4,3',4'-tetrol; peak 6) via idoxanthin (3,3',4'-trihydroxy-β,β-caroten-4-one; peak 5); **(c)** reduction of the single keto group in 4-ketolutein (peak 7, prepared from zygospores; no reference pigment available) yielded 4-hydroxylutein (peak 8). The on-line spectra of the products isozeaxanthin (peak 3) and crustaxanthin (peak 6) displayed absorbance maxima and spectral fine structure similar to zeaxanthin/β-carotene, while the on-line spectrum of the product 4-hydroxylutein (peak 8) had absorbance maxima and spectral fine structure similar to lutein/α-carotene.

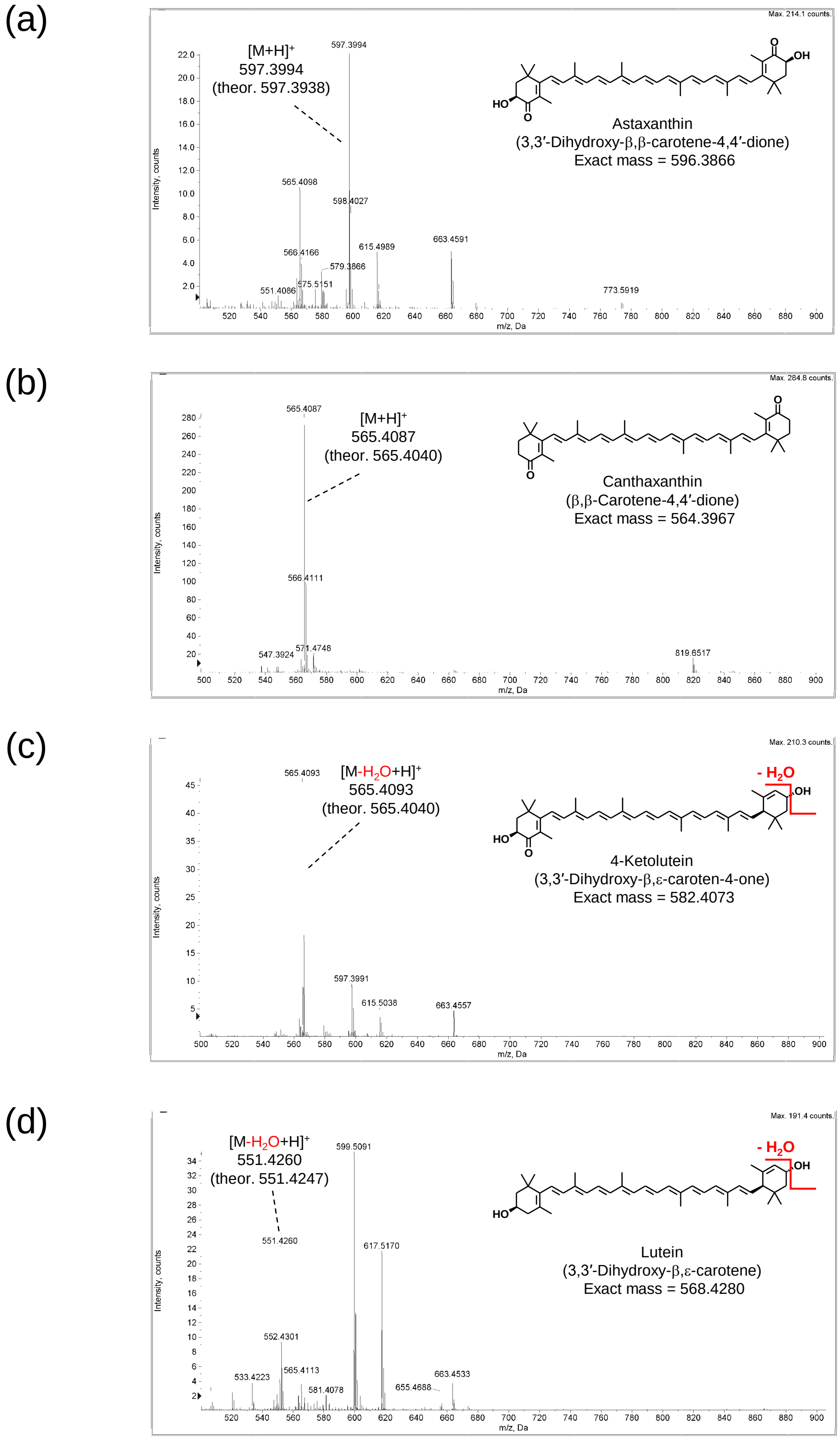

Fig. S3: Mass-spectrometric identification of ketocarotenoids extracted from zygospores of *C. reinhardtii.* LC-MS scans (APCI, positive ion mode) of the peaks identified as **(a)** astaxanthin, **(b)** canthaxanthin, and **(c)** 4-ketolutein, and of **(d)** lutein that served as reference pigment. For 4-ketolutein and lutein, the major ion species corresponded to the $[M-18+1]^+$ molecule; the abstraction of a water molecule in APCI-MS has been described repeatedly as a diagnostic feature of ε-ionone rings with a hydroxyl group at C3 (Aman et al., 2005, van Breemen et al., 1996, Dachtler et al., 2001).

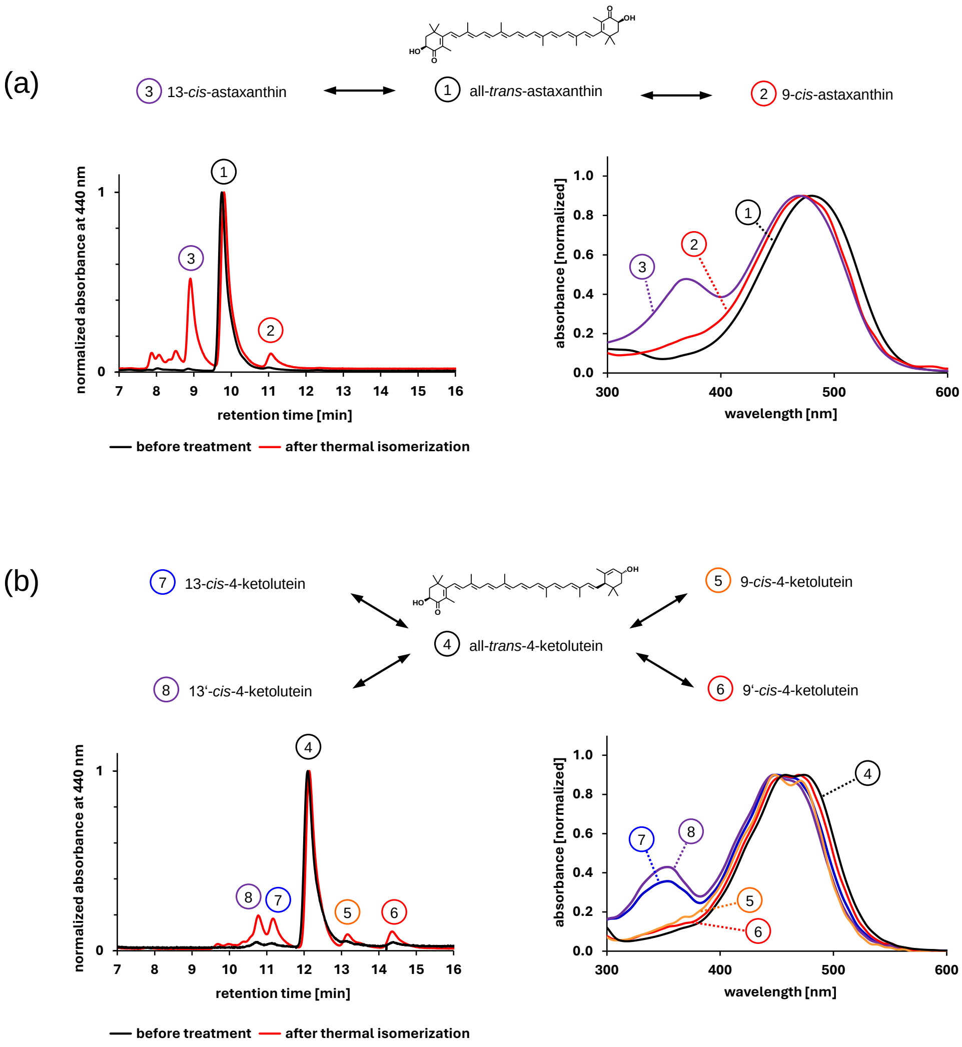

Fig. S4: HPLC-online-absorbance spectra (Gradient IV) of different isomers of **(a)** astaxanthin and **(b)** 4-ketolutein generated by thermal isomerization in toluene at 105°C for 15 min.

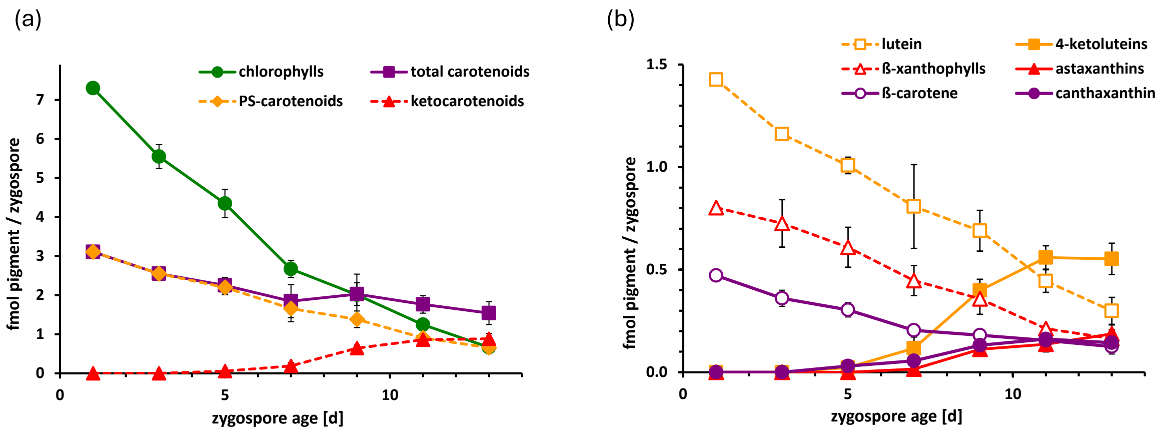

Fig. S5: (a) Time course of decline in photosynthetic pigments and the parallel accumulation of ketocarotenoids in samples from the zygospores of *C. reinhardtii* whose ultrastructural changes during 12 d maturation in the dark (starting after 1 d in light) at 22°C were investigated by TEM and by staining of lipids and polysaccharides (Fig. 3a). PS-carotenoids denote carotenoid species that were already present in vegetative (photosynthetic) cells (see Fig. 1). **(b)** Time course of changes in the individual precursors and their ketocarotenoid products. 4-Ketoluteins and astaxanthins comprise the free ketocarotenoids and their respective acyl esters, while β-xanthophylls include violaxanthin, antheraxanthin and zeaxanthin (but not 9'-*cis*-neoxanthin that likely is no precursor of astaxanthin). Per time point, mean values of results from two independent samples per zygote plate are shown (error bars denote upper and lower value); for day 1, only 1 sample was analyzed.

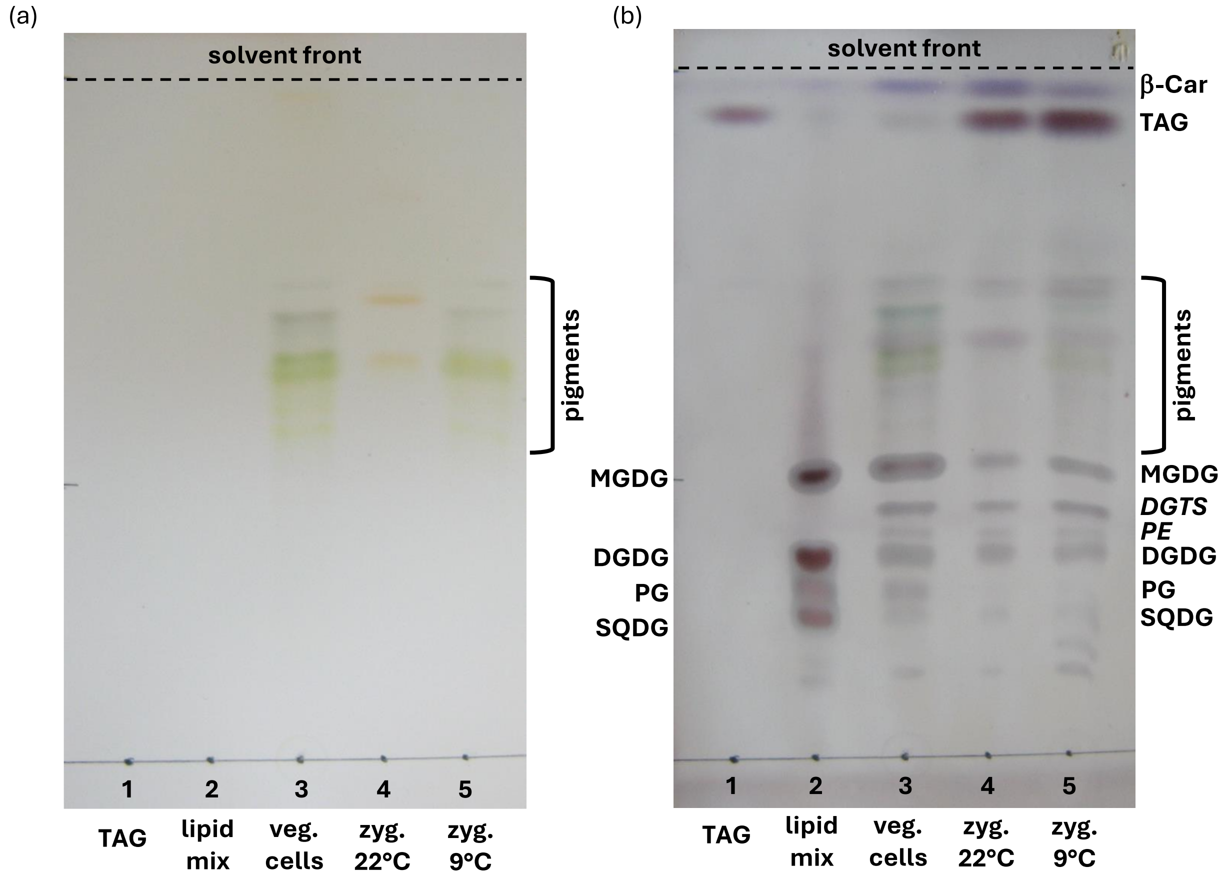

Fig. S6: TLC analysis of lipid classes in total lipid extracts from vegetative cells (lane 3) and from zygospores of *C. reinhardtii* matured at either 22°C (lane 4) or 9°C (lane 5) for 98 d. TLC plate **(a)** before and **(b)** after lipid staining. The colored bands in the "pigments" zone were already visible before lipid staining; they correspond to the chlorophylls and carotenoids detected by HPLC in Fig. 3b and were not analyzed in detail. The amount of lipid extract applied to each lane equaled 8 x 10^4 cells. The lipid standard mix applied to line 2 contained 1 µg of each lipid, the TAG standard (lane 1) was commercial cold-pressed sunflower oil with 0.6 µg TAG applied to the plate. Abbreviations of lipids are: DGDG, digalactosyldiacylglycerol; DGTS, l,2-diacylglyceryl-3-*O*-4'-(N,N,N-trimethyl)-homoserine; MGDG, monogalactosyldiacylglycerol; PE, phosphatidylethanolamine; PG, phosphatidylglycerol; SQDG, sulfoquinovosyldiacylglycerol; TAG, triacylglycerol. DGTS and PE (italicized) were tentatively identified based on Grünewald et al. (2001).

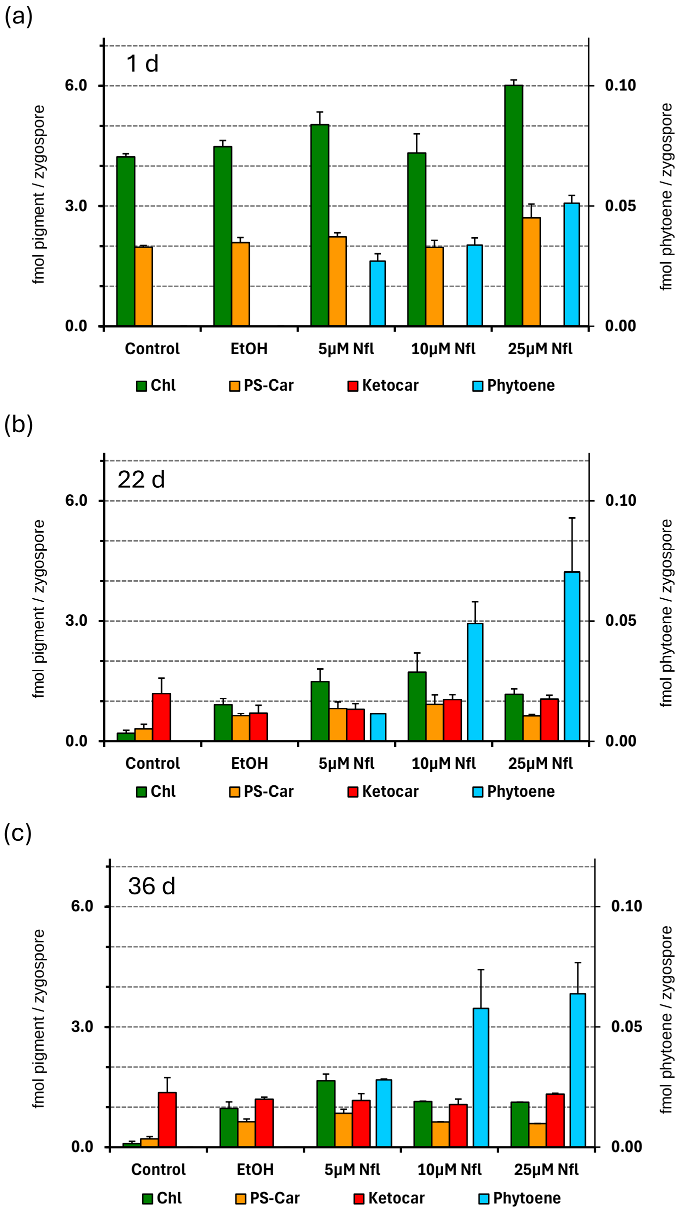

Fig. S7: Ketocarotenoid accumulation in zygospores maturing on TAP plates treated with three different concentrations of the phytoene desaturase inhibitor norflurazon (Nfl) dissolved in ethanol versus zygospores on untreated plates (Control) and zygospores on plates containing 0.7% (v/v) ethanol (EtOH). Pigment concentrations in zygospores **(a)** after 24 h light treatment right before dark incubation, **(b)** after dark incubation for 21 d, and **(c)** after dark incubation for 35 d. Phytoene concentrations are plotted on the right y-axis, concentrations of other pigments on the left y-axis. For each treatment, zygospores from two plates were analyzed separately and averages shown as columns (bars indicate the higher value of each data pair).

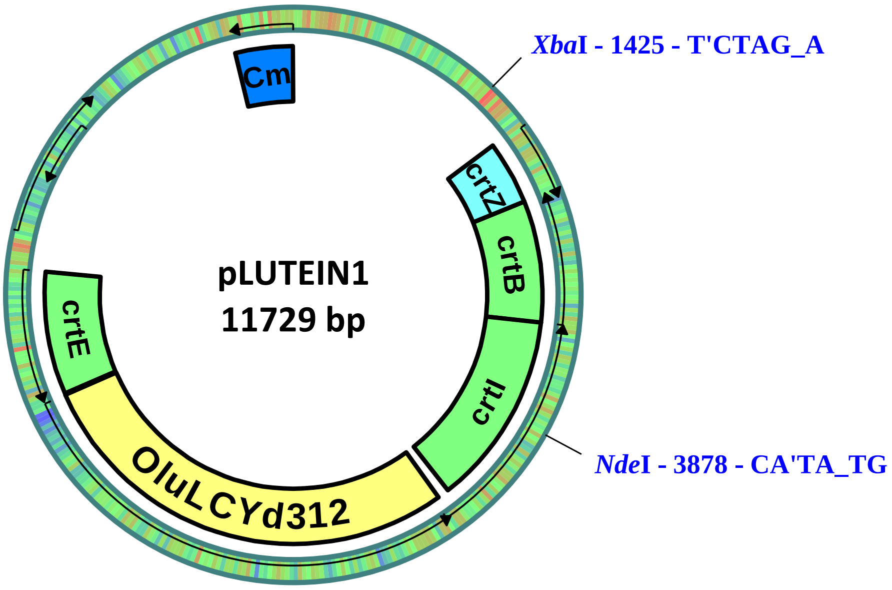

Fig. S8: Vector map of plasmid pLUTEIN1 that induces the accumulation of lutein, 3'-oxolutein, zeinoxanthin and zeaxanthin in *E. coli*. Gene abbreviatons are: Cm, chloramphenicol resistance; crtB, 15-*cis*-phytoene synthase; crtE, geranylgeranyl diphosphate synthase; crtI, phytoene desaturase; crtZ, carotene hydroxylase (all crt-genes are from the bacterium *Pantoea ananatis*); OluLCYd312, gene encoding the lycopene ε-cyclase/lycopene β-cyclase/light-harvesting complex (LHC) fusion protein from *Ostreococcus lucimarinus* with C-terminus shortened by the last 312 amino acids comprising the LHC domain.

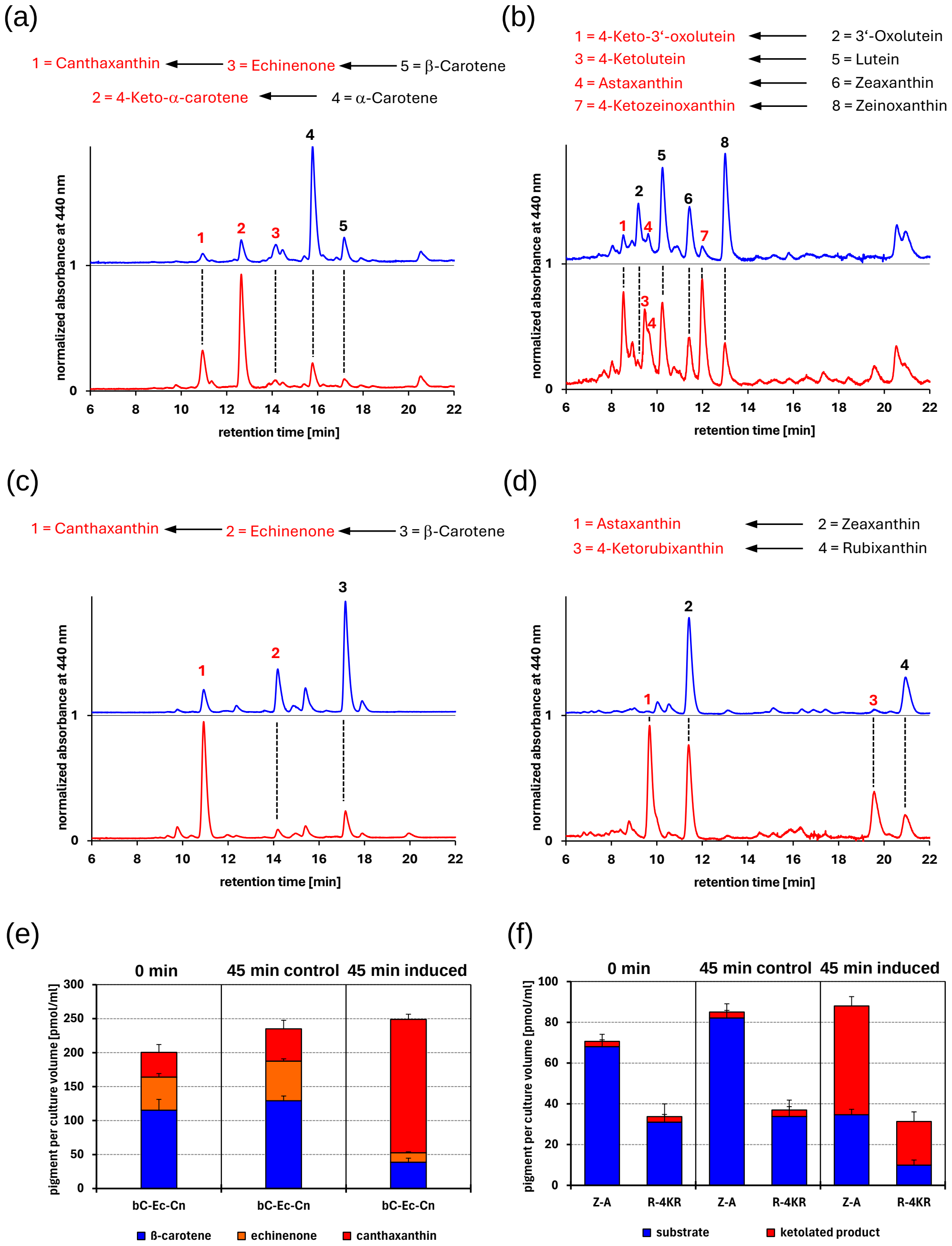

Fig. S9: Activity of BKT from *C. reinhardtii* after heterologous expression in *E. coli* strains engineered to supply different carotenoids as substrates. **(a-d)** HPLC chromatograms (Gradient IV) of pigment extracts from *E. coli* co-transformed with plasmids pBAD-CrBKT and **(a)** pALPHA1 yielding α- and β-carotene, **(b)** pLUTEIN1 yielding lutein, 3'-oxolutein, zeinoxanthin and zeaxanthin, **(c)** pACCAR25ΔcrtXZ yielding β-carotene, and **(d)** pACCAR25ΔcrtX yielding zeaxanthin and rubixanthin as potential substrates, before induction (blue traces) and after 45 min induction of CrBKT expression with 0.04% arabinose (red traces). **(e-f)** Carotenoid concentrations in bacterial suspensions of *E. coli* co-transformed with pBAD-CrBKT and either **(e)** pACCAR25ΔcrtXZ or **(f)** pACCAR25ΔcrtX, at the start of induction (0 min) and after 45 min of growth in the absence (45 min control) or presence (45 min induced) of 0.04% arabinose. Abbreviations of pigments are: A, astaxanthin; bC, β-carotene; Cn, canthaxanthin; Ec, echinenone; R, rubixanthin; 4-KR, 4-ketorubixanthin Z, zeaxanthin. Data are average values of 4 biological replicates with error bars denoting standard deviations.

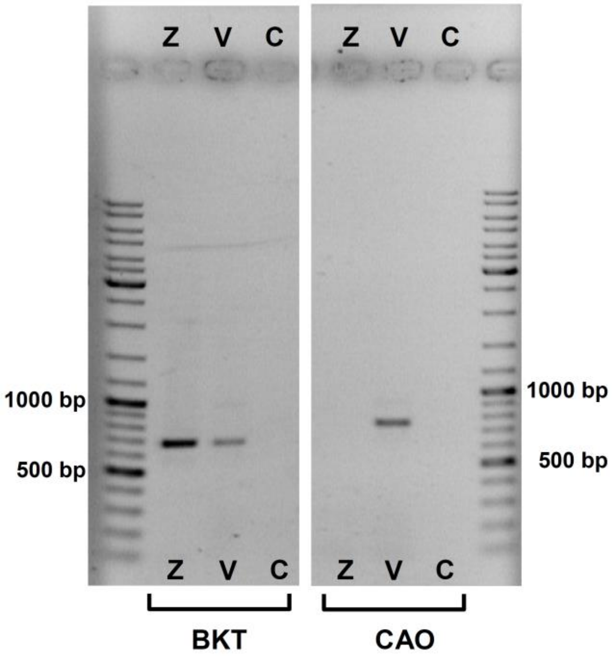

Fig. S10: Result of reverse-transcriptase PCR using total RNA prepared from 5 d old zygospores (Z) or from vegetative cells (V) of *C. reinhardtii*; pure water (C) was used as a control. Note that for analysis of vegetative cells, the amount of total RNA used (700 ng) was three times higher than for analysis of zygospores (230 ng). “BKT” designates PCR reactions using primers specific for β-carotene ketolase, “CAO” denotes reactions containing primers specific for chlorophyllide *a* oxygenase. PCR products were separated by agarose gel electrophoresis, stained with ethidium bromide, and the result documented as color-inverted black-and-white images. Expected product lengths were 688 bp for BKT and 765 bp for CAO. The GeneRuler™ DNA Ladder Mix (Fermentas) was used as a size marker.

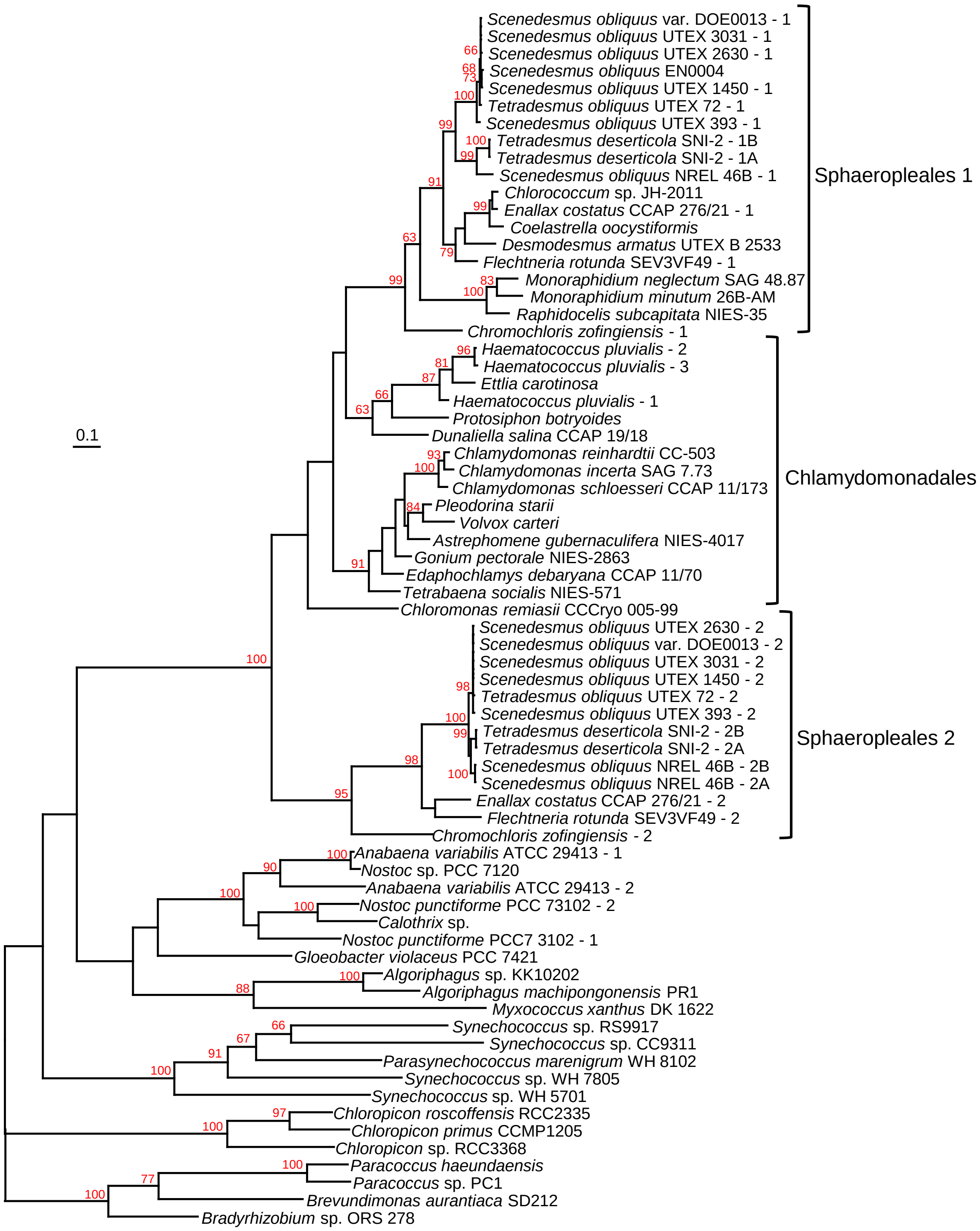

Fig. S11: Phylogeny of BKT proteins from green algae and CrtW proteins from selected cyanobacteria and other eubacteria. The maximum-likelihood tree was inferred from an alignment of 70 protein sequences from 52 species and encompassing 245 amino acid positions and was rooted to a CrtW protein cluster from α-proteobacteria. Bootstrap values (in red) are indicated for nodes with bootstrap support (100 replicates) ≥ 60%. See Table S2 for sequence accessions.

Figure S12 – 1/6

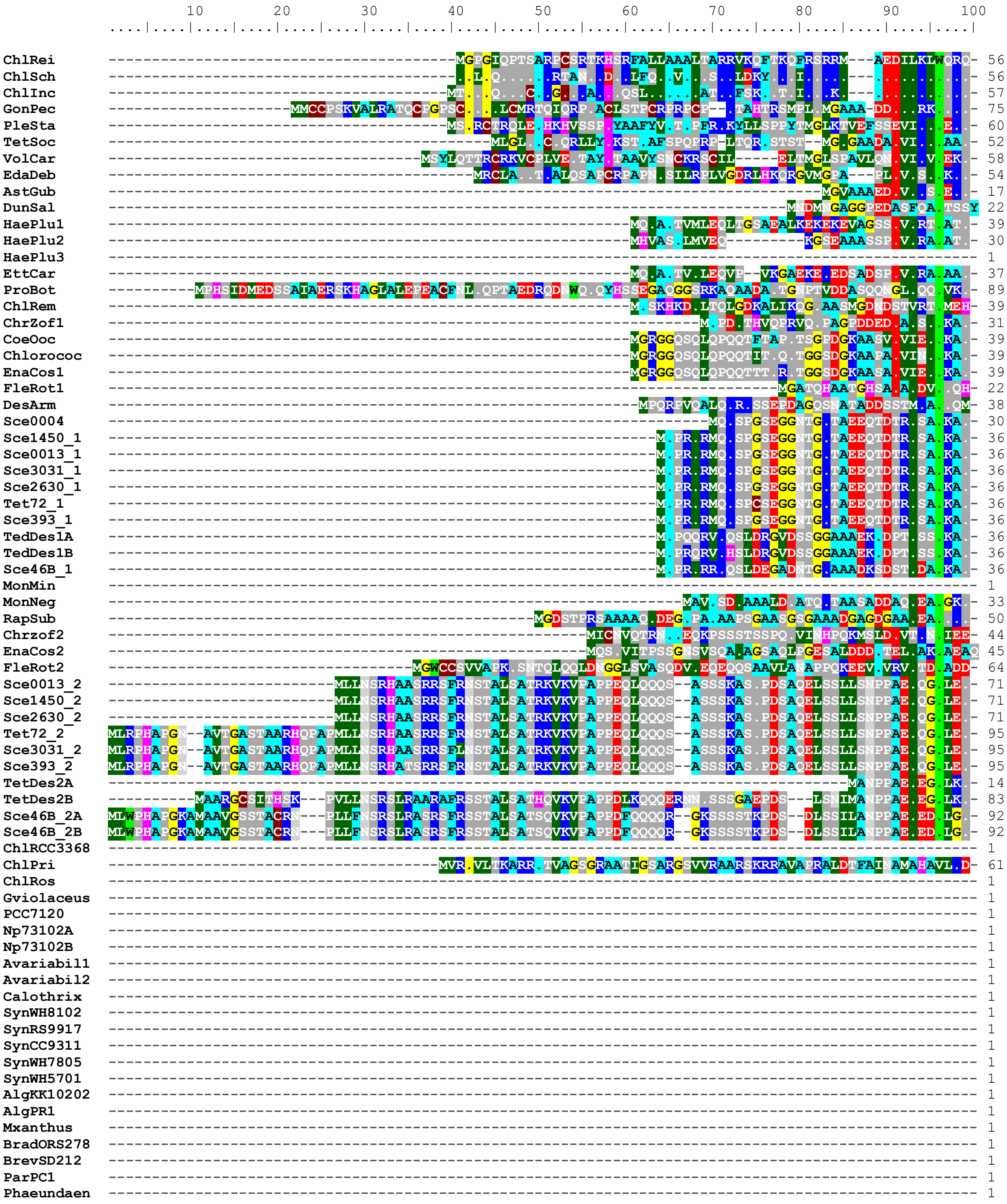

Figure S12 – 2/6

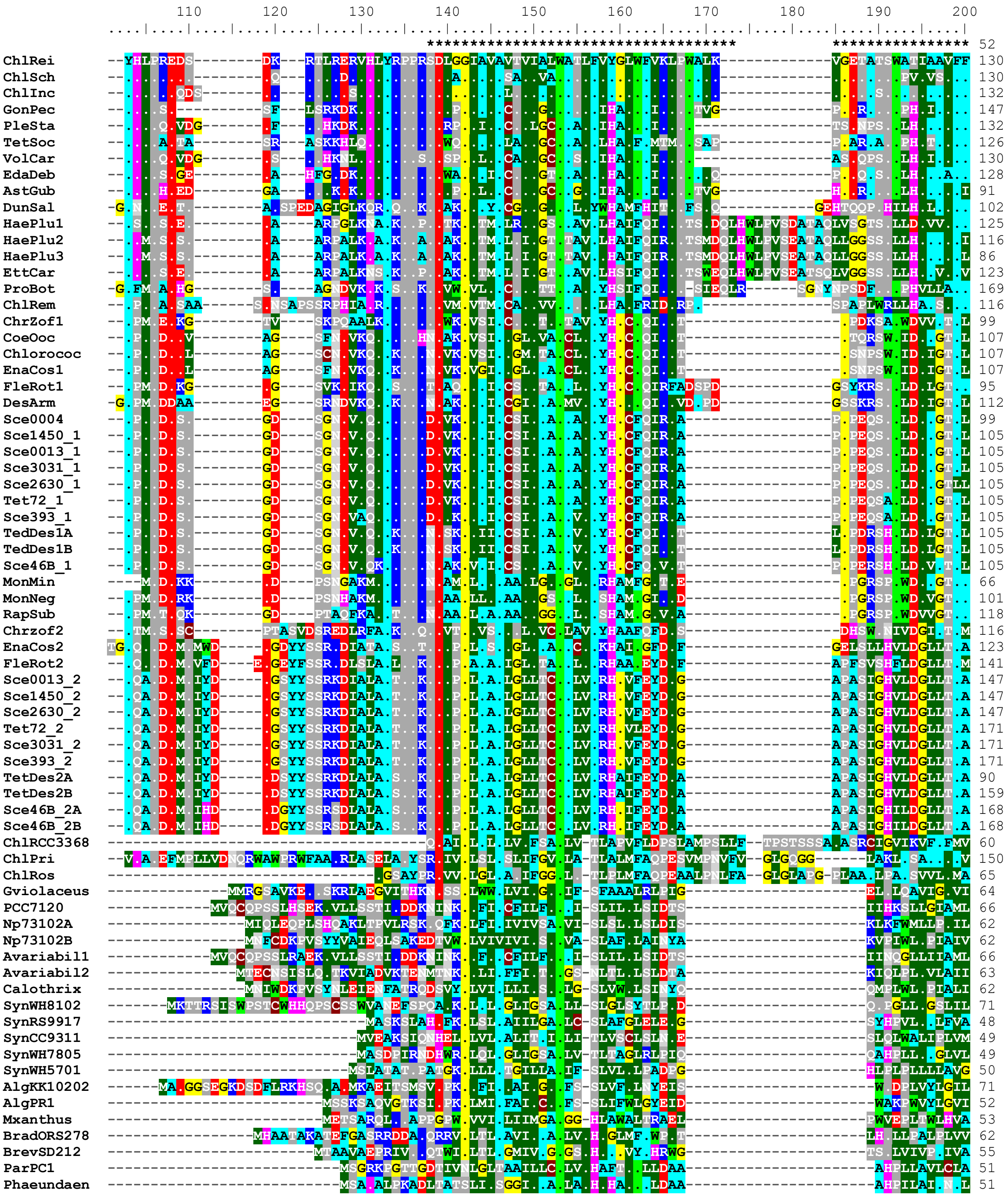

Figure S12 – 3/6

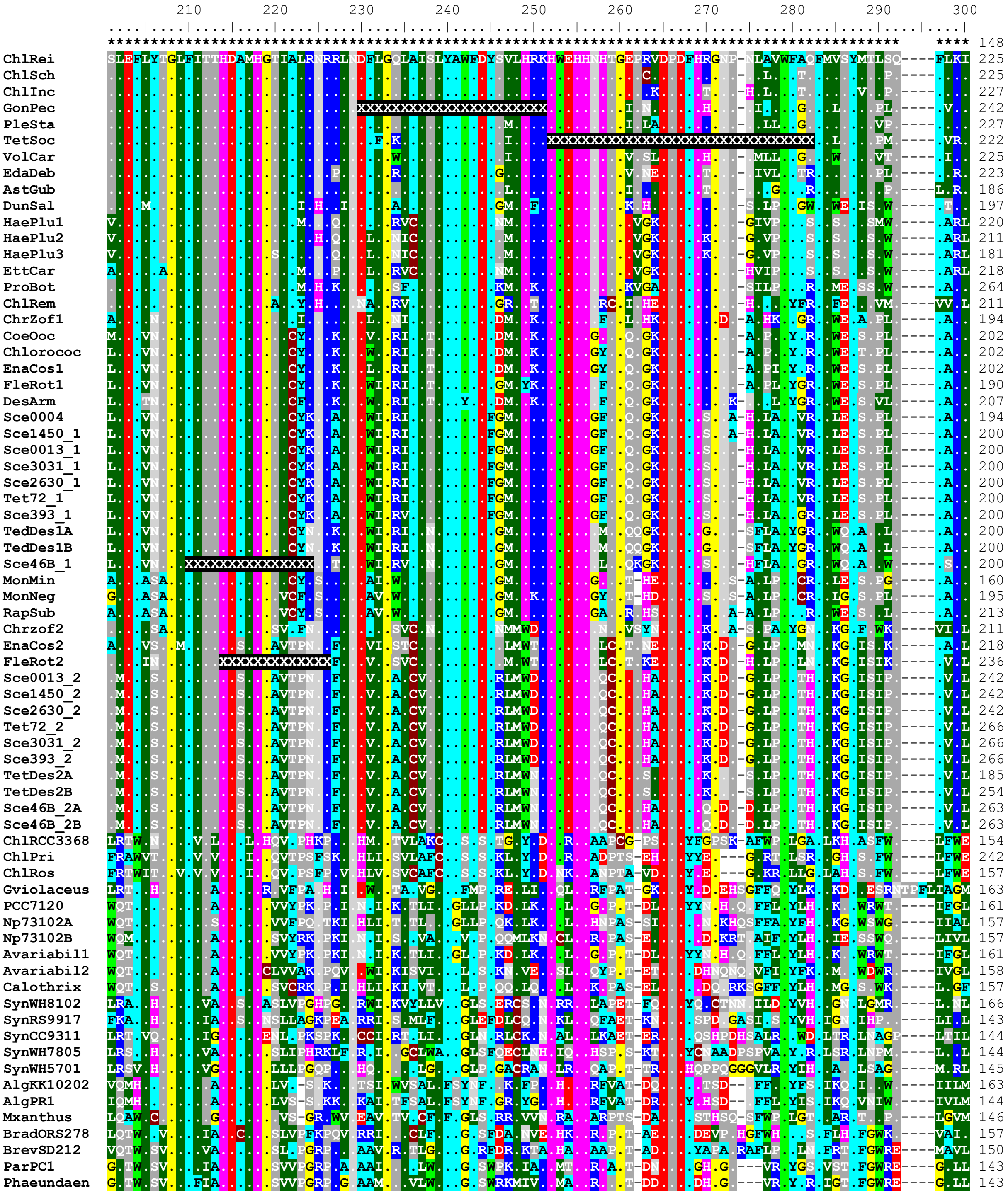

Figure S12 – 4/6

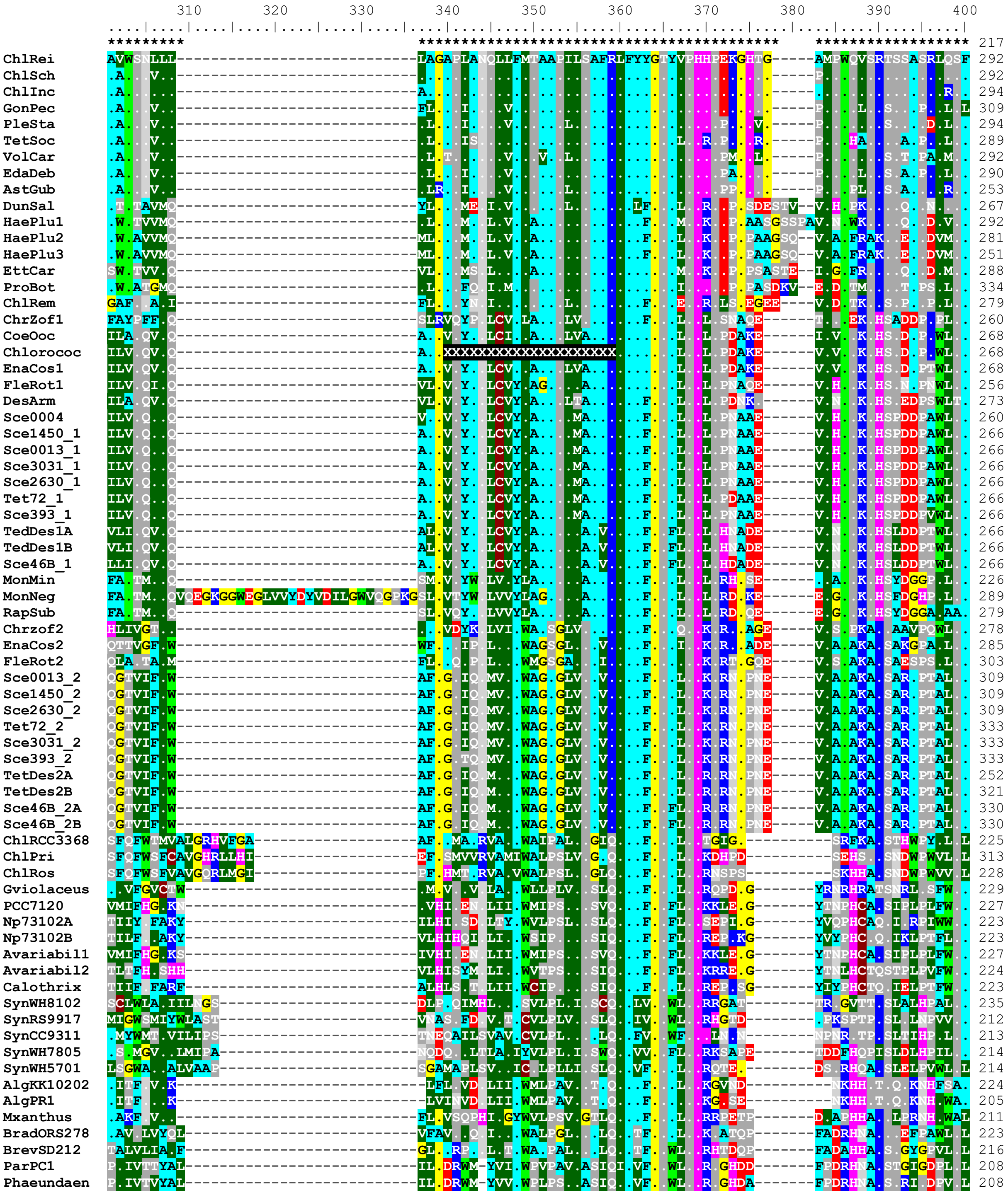

Figure S12 – 5/6

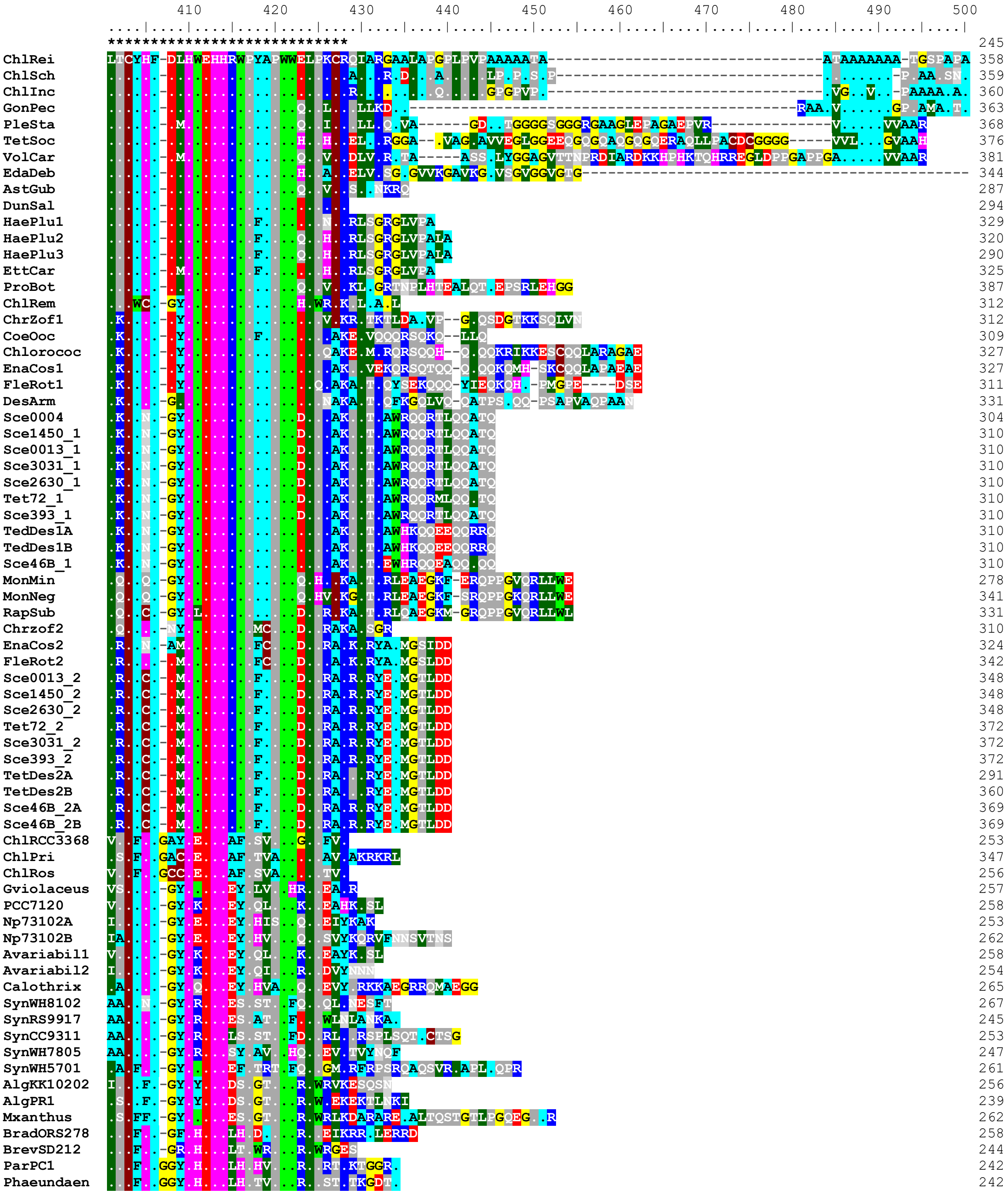

Figure S12 – 6/6

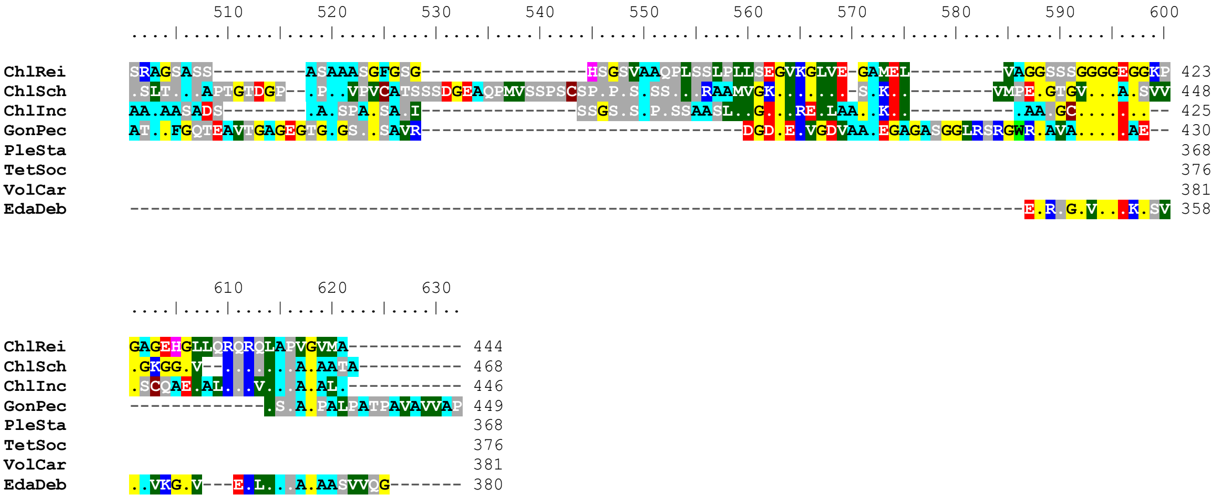

Fig. S12: Protein sequence alignment of BKT from algae and CrtW from selected cyanobacteria and other eubacteria. Using the sequence from *C. reinhardtii* (ChlrRei) as standard, identical amino acid residues in other sequences are plotted as dots. The alignment contains 70 sequences from 52 species and was generated by MAFFT v7.511 (Katoh et al., 2019) and subsequent manual editing. A truncated version of the alignment including only the amino acid positions labeled with asterisks above the first sequence was used for inference of the ML tree in Fig. 5 and Fig. S11. See Table S2 for sequence identities and accessions.

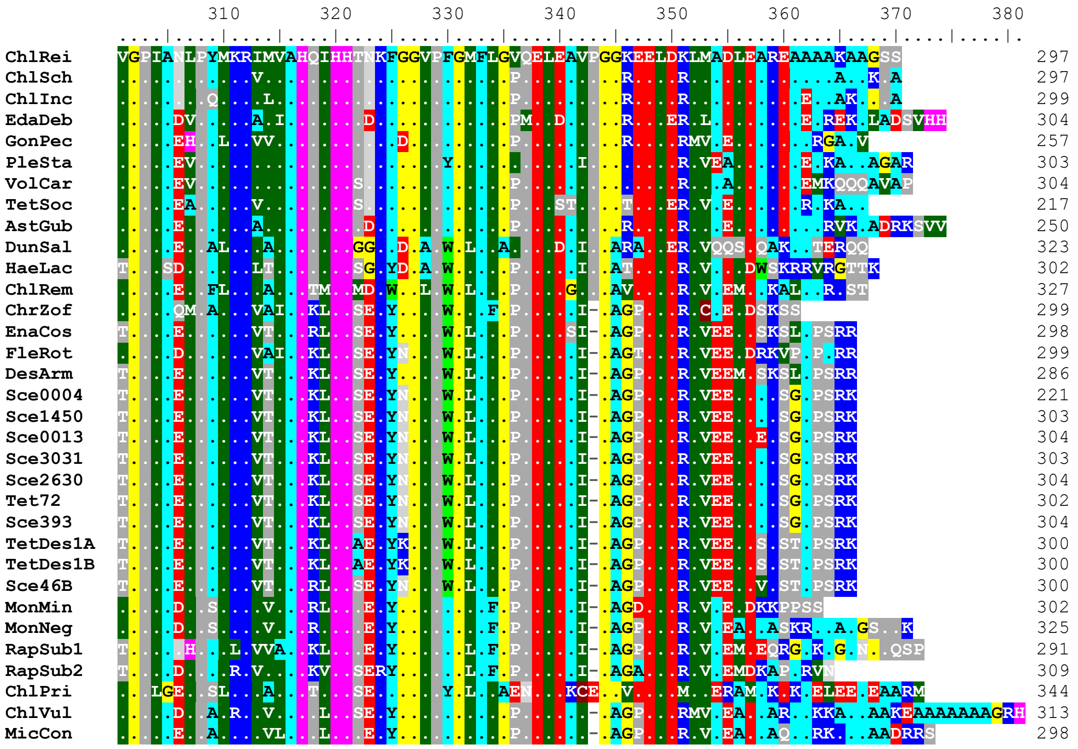

Fig. S13: Partial protein sequence alignment showing the C-termini of CYHB enzymes from green algae for which the BKT sequences in Fig. S12 were identified in publicly accessible databases. Using the sequence from *C. reinhardtii* (ChlrRei) as standard, identical amino acid residues in other sequences are plotted as dots. The alignment contains 33 sequences from 31 species and was generated by MAFFT v7.511 (Katoh et al., 2019) and subsequent manual editing. See Table S2 for sequence identities and accessions.
