# Supplementary material for: Chlamydomonas reinhardtii, Volvox carteri and related green algae accumulate ketocarotenoids not in vegetative cells but in zygospores
Source: Plant J. 2025 Feb 9;121(3):e17261. doi: 10.1111/tpj.17261 (PMC11808291; doi:10.1111/tpj.17261)
Supplement: Supplementary file 2 — Table S1. Identification of major free ketocarotenoids in pigment extracts from mature zygospores of Chlamydomonas reinhardtii. Table S2. Accessions of BKT/CrtW sequences in alignment of Figure S12 (used to infer the ML tree in Figure 5 and Figure S11) and of CHYB in Figure S13. [file TPJ-121-0-s002.pdf]

Table S1: Identification of major free ketocarotenoids in pigment extracts from mature zygospores of *C. reinhardtii*

Pigment	Rt [min]	absorbance maxima [nm]			III/II ratio	Pigment standard	APCI-MS mol. wt.	APCI-MS ion species	theoretical mol. wt.
Astaxanthin	8.7	—	—	479	—	+	597.3994	$[M+1]^+$	597.3938
Astaxanthin red.		—	*453*	*481*	*0.41*	+			
4-Ketolutein	9.7	—	456	472	—	n. a.	565.4093	$[M-18+1]^+$	565.4040
4-Ketolutein red.		*(425)*	*448*	*476*	*0.70*	n. a.			
Lutein (control)	10.8	(427)	448	477	0.67	+	551.4260	$[M-18+1]^+$	551.4247
Canthaxanthin	12.9	—	—	478	—	+	565.4087	$[M+1]^+$	565.4040
Canthaxanthin red.		—	*454*	*481*	*0.38*	+			

Data for ketocarotenoids after reduction with $NaBH_4$ are italicized. „n. a.“, no pigment standard available.

Table S2: Accessions of BKT/CrtW sequences in alignment of Fig. S12 (used to infer the ML tree in Figs. 5 and S11) and of CHYB in Fig. S13

Order	Family	Species	BKT - Abbrev. in Fig. S12:<br>Accession Phytozome or GenBank	CHYB - Abbrev. in Fig. S13:<br>Accession Phytozome or GenBank
Sphaeropleales	Chromochloridaceae	*Chromochloris zofingiensis* SAG 211-14 v5.0	ChrZof1: Chrzof1|3917 ; AAV41371	ChrZof: ABS50237.1
			ChrZof2: Chrzof1|9675 ; QSH48345.1	
	Scenedesmaceae	*Desmodesmus armatus* UTEX B 2533 v2.0	DesArm: DesarB2533_2|184029	DesArm: DesarB2533_2|198727
		Scenedesmus sp. NREL 46B-D3 v1.0	Sce46B_1: Scesp_1|1680266	Sce46B: Scesp_1|1477468
			Sce46B_2A: Scesp_1|1290392	
			Sce46B_2B: Scesp_1|1298489	
		Scenedesmus obliquus var. DOE0013 v1.0	Sce0013_2: SceoblDOE13_1|1043796	Sce0013: SceoblDOE13_1|451800
			Sce0013_1: SceoblDOE13_1|450043	
		Scenedesmus obliquus EN0004 v1.0	Sce0004: SceoblEN4_1|342204	Sce0004: SceoblEN4_1|619393
		Scenedesmus obliquus UTEX 393 v2.0	Sce393_1: Sceobl393_2|1761809	Sce393: Sceobl393_2|1724531
			Sce393_2: Sceobl393_2|1970470	
		Scenedesmus obliquus UTEX 3031	Sce3031_1: Sceob152z_1|22870	Sce3031: Sceob152z_1|28322
			Sce3031_2: Sceob152z_1|28590	
		Scenedesmus obliquus var. UTEX 1450 v1.0	Sce1450_1: Sceobl1450_1|971985	Sce1450: Sceobl1450_1|1306676
			Sce1450_2: Sceobl1450_1|956000	
		Scenedesmus obliquus var. UTEX2630 v1.0	Sce2630_1: Sceobl2630_1|806443	Sce2630: Sceobl2630_1|1137783
			Sce2630_2: Sceobl2630_1|801591	
		Tetradesmus deserticola SNI-2 v1.1	TetDes2A: TetrdesSNI2_1|7698476	TetDes1A: TetrdesSNI2_1|7704952
			TetDes2B: TetrdesSNI2_1|7717465	TetDes1B: TetrdesSNI2_1|7724217
			TedDes1A: TetrdesSNI2_1|7474963	
			TedDes1B: TetrdesSNI2_1|7552627	
		Tetradesmus obliquus UTEX 72 v1.1	Tet72_1: Tetrobl72_1|3669810	Tet72: Tetrobl72_1|3870562
			Tet72_2: Tetrobl72_1|3824621	
		Flechtneria rotunda SEV3VF49 v1.1	FleRot1: Flerot1_1|11185236	FleRot: Flerot1_1|11343763
			FleRot2: Flerot1_1|11578359	
		Enallax costatus CCAP 276/31 v1.1	EnaCos1: Enacos1_1|5757247	EnaCos: Enacos1_1|6537931
			EnaCos2: Enacos1_1|6640017	

		Coelastrella oocystiformis	CoeOoc: *AEM45622*	
	Selenastraceae	*Monoraphidium minutum* 26B-AM v1.0	MonMin: Monmin1|415167	MonMin: Monmin1|380629 ; *KAI8464055.1*
		Monoraphidium neglectum SAG 48.87	MonNeg: Monneg1|15159	MonNeg: Monneg1|1505 (N-term. extended)
		Raphidocelis subcapitata NIES-35	RapSub: Rapsub1_1|32	RapSub1: GBF99089.1
				RapSub2: GBF98825.1
Chlamydomonadales	Chlamydomonadaceae	*Chlamydomonas reinhardtii* CC-503 v5.6	ChlRei: Chlre5_6|13221 ; *Q4VKB4.1*	ChlRei: Chlre5_6|13220 ; *XP_001698698.1*
		Chlamydomonas incerta SAG 7.73	ChlInc: Chlin1|11187	ChlInc: Chlin1|11186 ; *KAG2426606.1*
		Chlamydomonas schloesseri CCAP 11/173	ChlSch: Chlsc1|13447	ChlSch: Chlsc1|13448 ; *KAG2432680.1*
		Chloromonas remiasii CCCryo 005-99 v1.0	ChlRem: Chlremi1|2423028	ChlRem: Chlremi1|2465423
	Volvocaceae	*Volvox carteri* v2.1	VolCar: Volca2_1|7425	VolCar: Volca2_1|7440 ; *XP_002957595.1*
		Pleodorina starrii NIES-1363	PleSta: PLESTM_001306400 ; *GLC42230.1*	PleSta: PLESTM_001304600 ; *GLC42214.1*
	Goniaceae	*Gonium pectorale* NIES-2863	GonPec: Gonpec1|scaffold_753:5622-10331 + Gonpec1| scaffold_295:28166-28573	GonPec: Gonpec1|11812 ; *KXZ45494.1*
		Astrephomene gubernaculifera NIES-4017	AstGub: Astgub1|4443	AstGub: assembled from SRA data of project SRX10107834, run SRR13719267
	incertae sedis	*Edaphochlamys debaryana* CCAP 11/70	EdaDeb: Edade1|12908	EdaDeb: *KAG2482852.1*
	Tetrabaenaceae	*Tetrabaena socialis* NIES-571	TetSoc: Tetso1|12822 (partial due to large gap)	TetSoc: assembled from SRA data of project SRX3367147, run SRR6260811
	Dunaliellaceae	*Dunaliella salina* CCAP19/18	DunSal: Dunsal1_1|2573 ; *KAF5838589*	DunSal: Dunsal1_1|10288 ; *KAF5833419.1*
	Chlorococcaceae	*Chlorococcum* sp. JH-2011	Chlorococ: *AEM45623*	
	Protosiphonaceae	*Protosiphon botryoides*	ProBot: *AEM45621*	
	Haematococcaceae	*Haematococcus pluvialis*	HaePlu1: *CAA60478*	HaeLac: *KAJ9521810.1*
			HaePlu2: *BAA08300*	
			HaePlu3: *ABB70497*	
		Ettlia carotinosa	EttCar: *AEM45620*	
Trebouxiophyceae / Chlorellales	Chlorellaceae	*Chlorella vulgaris*	---	ChlVul: *KAI3425075.1*
		Micractinium conductrix	---	MicCon: *PSC69401.1*
Chloropicophycaea / Chloropicales	Chloropicaceae	*Chloropicon primus* CCMP1205	ChlPri: Chlpri1|6394 ; *QDZ23876.1*	ChlPri: *QDZ19102.1*
		Chloropicon roscoffensis RCC2335	ChlRos: CAMPEP_0197513860 ; *WZN65237.1*	
		Chloropicon sp. RCC3368	ChlRCC3368: CAMPEP_0198246870	

Cyanobacteria 1		*Anabaena variabilis* ATCC 29413	Avariabil1: *WP_011320583.1*	
			Avariabil2: *WP_011318840.1*	
		Calothrix sp.	Calothrix: *WP_190539215.1*	
		Gloeobacter violaceus PCC 7421	GloVio: *BAC89669.1*	
		Nostoc punctiforme PCC 73102	NP73102A: *WP_012411103.1*	
			NP73102B: *WP_012412154.1*	
		Nostoc sp. PCC 7120	PCC7120: *WP_010997340.1*	
Cyanobacteria 2		*Synechococcus* sp. CC9311	SynCC9311: *ABI46516.1*	
		Synechococcus sp. RS9917	SynRS9917: *WP_007100135.1*	
		Synechococcus sp. WH 5701	SynWH5701: *WP_006173021.1*	
		Synechococcus sp. WH 7805	SynWH7805: *WP_006041055.1*	
		Parasynechococcus marenigrum WH 8102	SynWH8102: *WP_011128232.1*	
Other bacteria		*Algoriphagus* sp. KK10202	AlgKK10202: *ABB88952.1*	
		Algoriphagus machipongonensis PR1	AlgPR1: *WP_008197705.1*	
		Myxococcus xanthus DK 1622	Mxanthus: *WP_201422169.1*	
		Bradyrhizobium sp. ORS 278	BradORS278: *WP_012029995.1*	
		Brevundimonas aurantiaca SD212	BrevSD212: *WP_336970560.1*	
		Paracoccus haeundaensis	Phaeundaen: *AAY28417.1*	
		Paracoccus sp. PC1	ParPC1: *Q44261.1*	
